# Neural similarity between mentalizing and live social interaction during the transition to adolescence

**DOI:** 10.1002/hbm.25903

**Published:** 2022-05-12

**Authors:** Junaid S. Merchant, Diana Alkire, Elizabeth Redcay

**Affiliations:** ^1^ Neuroscience and Cognitive Science Program University of Maryland College Park Maryland USA; ^2^ Department of Psychology University of Maryland College Park Maryland USA

**Keywords:** development, fMRI, mentalizing, social cognition, social interaction

## Abstract

Social interactions are essential for human development, yet little neuroimaging research has examined their underlying neurocognitive mechanisms using socially interactive paradigms during childhood and adolescence. Recent neuroimaging research has revealed activity in the mentalizing network when children engage with a live social partner, even when mentalizing is not required. While this finding suggests that social‐interactive contexts may spontaneously engage mentalizing, it is not a direct test of how similarly the brain responds to these two contexts. The current study used representational similarity analysis on data from 8‐ to 14‐year‐olds who made mental and nonmental judgments about an abstract character and a live interaction partner during fMRI. A within‐subject, 2 (Mental/Nonmental) × 2 (Peer/Character) design enabled us to examine response pattern similarity between conditions, and estimate fit to three conceptual models of how the two contexts relate: (1) social interaction and mentalizing about an abstract character are represented similarly; (2) interactive peers and abstract characters are represented differently regardless of the evaluation type; and (3) mental and nonmental states are represented dissimilarly regardless of target. We found that the temporal poles represent mentalizing and peer interactions similarly (Model 1), suggesting a neurocognitive link between the two in these regions. Much of the rest of the social brain exhibits different representations of interactive peers and abstract characters (Model 2). Our findings highlight the importance of studying social‐cognitive processes using interactive approaches, and the utility of pattern‐based analyses for understanding how social‐cognitive processes relate to each other.

## Graphical abstract

Social interactions are of fundamental importance to human life, but our understanding of the neurocognitive systems underlying this complex social behavior is relatively lacking. The present study used representational similarity analysis (RSA) to examine the similarity of response patterns between social interaction and mentalizing (i.e., the ability to make inferences about another's mental state), which is thought to be an important underlying process. We found that the clusters in the temporal lobe exhibit similar responses when an individual is making mental state inferences about an abstract character and when interacting with a peer, even when the interaction required no mentalizing, suggesting a neurocognitive link between the social interaction and mentalizing in these regions.

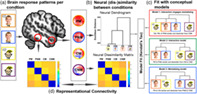

## 1 INTRODUCTION

Social interactions, or reciprocal exchanges between socially engaged individuals, are a ubiquitous part of daily life and play an important role in shaping the human brain. Despite the relative ease with which most individuals are able to engage in social interactions, a myriad of neurocognitive processes underlies this complex social behavior. Mentalizing (also known as “theory of mind”) is the early developing ability to attribute mental states to others that are thought to play an increasingly critical role in navigating social interactions from childhood into adulthood (Frith & Frith, 2001; Frith & Frith, 2012; Wellman, 2017). Neuroimaging research has revealed a common network of brain regions, often referred to as the “mentalizing network,” that are activated across a range of tasks that require mental state reasoning. This network includes the dorsal and ventral regions of the medial prefrontal cortex (dmPFC and vmPFC), inferior frontal gyrus (IFG), dorsolateral prefrontal cortex (dlPFC), precuneus, temporoparietal junction (TPJ), anterior temporal lobe (ATL), and superior temporal sulcus (STS; Mar, 2011; Molenberghs et al., 2016; Schurz et al., 2014). However, most neuroimaging investigations of mentalizing have utilized noninteractive experimental paradigms wherein adults make third‐person attributions about abstract characters or simply observe social behavior. Thus, relatively little is known about the neural substrates linking mentalizing to active engagement in social‐interactive contexts, much less the neurocognitive relationship between the two during the transition to adolescence, which is a period marked by significant social development.

Despite the relative lack of neuroimaging work examining the relationship between mentalizing and social interaction, evidence from developmental research suggests that the progression from childhood to adolescence may be an important period for understanding how mentalizing and peer interaction relate, as developmental changes are seen in both. Starting in early childhood, individuals' peer relationships become increasingly important, such that they spend progressively more time with friends over parents, and this social re‐orienting is accompanied by changes in brain systems associated with perception, motivation/affect, and executive function (Ladd, 1999; Nelson et al., 2016; Parker et al., 2006). This trajectory aligns with neuroimaging work demonstrating that the mentalizing network is functionally distinct by the age of three (Richardson et al., 2018), but exhibits increasing functional specialization through adolescence and into adulthood (Gweon et al., 2012; Moraczewski et al., 2020; Richardson et al., 2020). There is also increased sensitivity in the brain's reward system to social contexts as children transition into adolescence (Chein et al., 2011; Moreira & Telzer, 2018; Smith et al., 2015), and activity in the reward and mentalizing networks during adolescence is indicative of a tendency to spontaneously integrate peer perspectives into self‐evaluations (Jankowski et al., 2014; Pfeifer et al., 2009; Van der Cruijsen et al., 2019). In particular, this work suggests an inverted‐U‐shaped trajectory in the neural sensitivity to and self‐report of self‐conscious emotions (i.e., social emotions like embarrassment that indicate perceived evaluation by others) from middle childhood to young adulthood (Somerville et al., 2013). Together, these findings indicate that the pre‐adolescent to early adolescent period is a promising age‐range for understanding the neurocognitive links between mentalizing and social interaction, and how they develop with age. Until recently, however, most of our understanding of the mentalizing network comes from work on adults.

Neuroimaging studies using adult samples have demonstrated activations primarily within the mentalizing network across tasks that elicit mentalizing through a variety of noninteractive approaches. This includes tasks with explicit instructions to make inferences about an abstract character's mental state (compared to, for example, physical characteristics), as well as the assessment of *spontaneous* mentalizing—that is, elicited independently of task demands (i.e., without prompting)^1^—for example, when individuals use mental‐state language to describe the behavior of moving shapes that are animated to resemble agency (see Mar, 2011; Molenberghs et al., 2016; Schurz et al., 2014). The spatial convergence of brain activations across these varied experimental paradigms has been used as evidence for the mentalizing brain network and laid the foundations for much of the social cognitive neuroscience literature since. However, this foundation has a crucial limitation: it characterizes social cognition only in observational (i.e., noninteractive) contexts, leaving gaps in our understanding of the neurocognitive processes involved in real‐world, social‐interactive behavior.

## 2 SECOND‐PERSON NEUROSCIENCE

A body of neuroimaging studies, collectively referred to as “second‐person neuroscience,” has provided new avenues for understanding the neurocognitive processes involved in social interaction (Redcay & Schilbach, 2019; Schilbach et al., 2013). This set of approaches has utilized paradigms that involve engaging with a social partner in real time, thereby providing practical and theoretical advancements to the study of social cognition compared to traditional, third‐person approaches. For instance, second‐person neuroscience work using developmental samples has demonstrated that children are more motivated and rewarded when interacting with a live social partner compared to a computer or character, as indexed through behavioral responses, self‐report, and neural activations (Alkire et al., 2018; Rice et al., 2016; Rice & Redcay, 2016; Warnell et al., 2018). Second‐person neuroscience studies using adult and child samples have also demonstrated that simply engaging with a live social partner recruits a more extended network of brain systems than has been previously reported in traditional social neuroscience studies relying on noninteractive, third‐person approaches (Redcay et al., 2010; Redcay & Schilbach, 2019; Redcay & Warnell, 2018; Warnell et al., 2018). In particular, this work has demonstrated greater activations in the mentalizing network when individuals simply perceive a real‐life social partner (versus an abstract or unknown social entity), even in the absence of any explicit mental state information or task demands to mentalize (Alkire et al., 2018; Redcay & Schilbach, 2019; Warnell et al., 2018). Although these findings seem to indicate that individuals are spontaneously mentalizing in the presence of a social partner, it is possible that these brain regions are part of an integrated network of systems that support “online” social behavior (i.e., when actively engaged in real‐time social contexts) that have superficial overlap with regions supporting “offline” social cognition in noninteractive and observational contexts (Schilbach, 2014; Schilbach et al., 2013).

Attempts at interpreting the meaning of activations in the mentalizing network during social engagements point to the broader limitations of reversely inferring a cognitive process from the spatial location of brain activations (Hutzler, 2014; Poldrack, 2006). For instance, Alkire et al. (2018) reported overlapping activations in the ATL, STS, and IFG when children reasoned about the mental state of an abstract character *and* when they engaged in social interactions that did not explicitly require mental state reasoning, and inferred that social interaction may induce spontaneous mentalizing. However, these brain regions are associated with other, higher‐order cognitive processes that may be important for social interactions—the ATL and STS are associated with the representation of person‐specific information (Anzellotti, 2017; Anzellotti & Caramazza, 2017; Blank et al., 2015; Collins et al., 2016; Olson et al., 2013; Perrodin et al., 2015; Simmons et al., 2010; Wang et al., 2017), and the IFG is implicated in a range of executive processes, such as working memory and behavioral inhibition (Breitling et al., 2020; Drummond et al., 2017; Hartwigsen et al., 2019). Thus, it is unclear if the overlapping activity associated with social interaction reported by Alkire et al. (2018) is specific to mentalizing, or reflects other cognitive processes utilized within social interactive contexts.

Multi‐voxel, pattern‐based approaches help alleviate some of the problems of reverse inference (Hebart & Baker, 2018; Poldrack, 2011). In particular, representational similarity analysis (RSA) leverages voxel‐wise activity patterns to estimate the similarity of neural responses elicited by different task conditions, which can be used to infer commonalities in the underlying neurocognitive process (Kriegeskorte et al., 2008). RSA has proven to be a useful tool for assessing similarities between domain‐general and social‐cognitive functions (e.g., similar response patterns for social and physical distance in the inferior parietal lobule; Parkinson et al., 2014), and for disentangling subtle differences in response patterns across brain regions engaged by a common task that are indicative of diverging function (e.g., different trait dimensions represented across cortical midline structures during self–other judgments; Feng et al., 2018). Of interest, applications of this approach in adult samples have demonstrated that activity patterns in mentalizing regions maintain stable representations of mental states across targets (e.g., personally known versus abstract others; Weaverdyck et al., 2021), enable fine‐grained inferences of others' emotions (Skerry & Saxe, 2015), and are involved in learning social information about unknown others (Dziura & Thompson, 2018). Importantly, RSA has revealed that activity patterns in mentalizing regions of both adults and children distinguish mental‐state information and nonmental, social information, and that the distinctiveness of these patterns increased from 5 to 12 years of age, even when univariate activations do not exhibit a relationship with age (Richardson et al., 2020). Together, these findings demonstrate that RSA provides a sensitive approach to assess the similarity of neurocognitive processes associated with social interaction and mentalizing, and to assess how this relationship changed with age.

## 3 CURRENT STUDY

**FIGURE 1:**
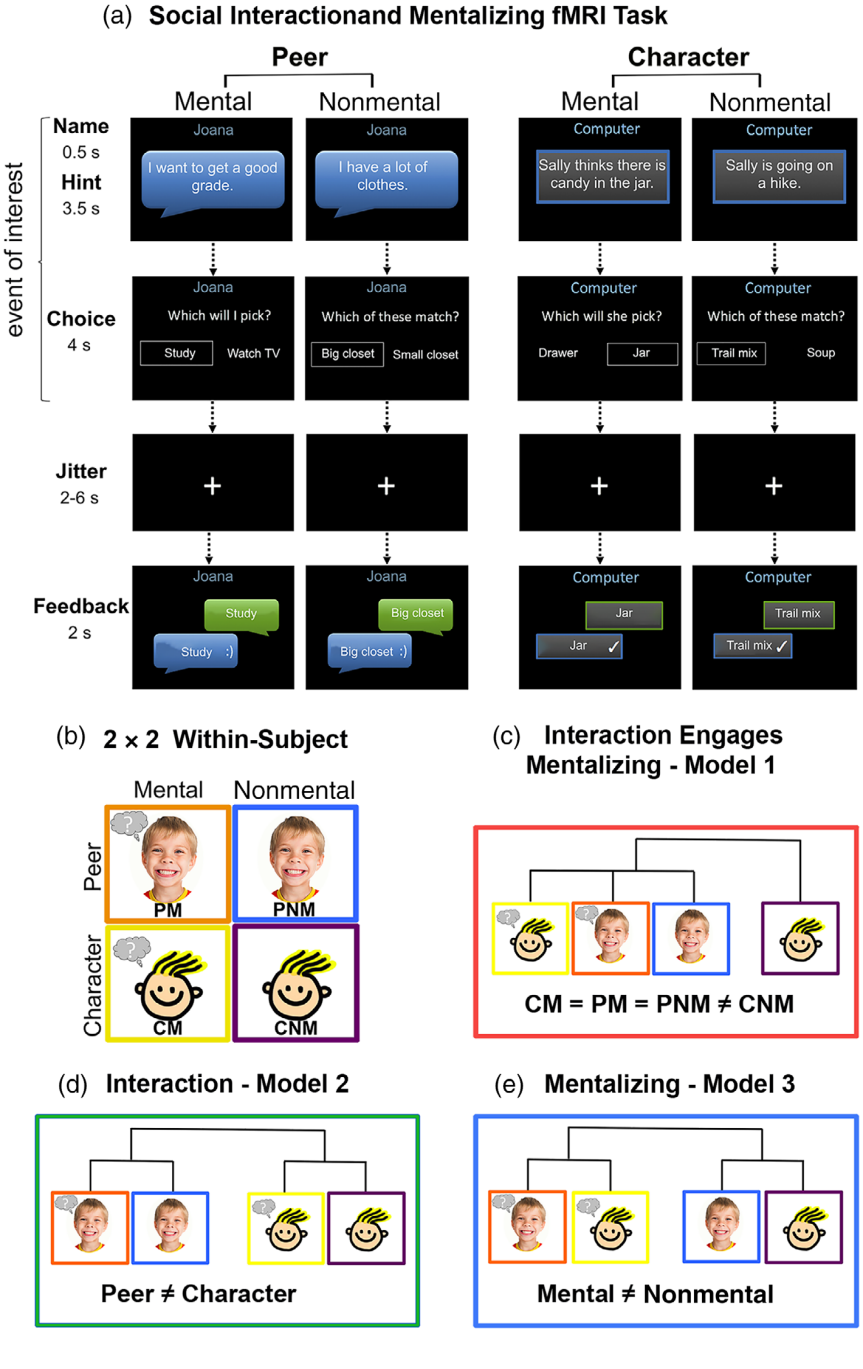
Schematic of the social‐interactive fMRI task (a). Participants were given a half‐second cue indicating if they would be answering questions provided by their interaction partner (peer) or answering questions presented by the computer about a story character (character). Hints were provided for 3.5 s and either required using mental state information about the target (mental), or nonmental, physical information (nonmental). This yielded a fully within‐subject, 2 (peer/character) × 2 (mental/nonmental) design (b). PM, peer mental; PNM, peer nonmental; CM, character mental, and CNM, character nonmental. Model 1 = interaction engages mentalizing (c), model 2 = interaction model (d), and model 3 = mentalizing model (e)

The overarching goal of the current study is to advance our understanding of the neuro‐representational links between social interaction and mentalizing during the transition to adolescence. In particular, the current work was motivated by three central questions: (1) Do brain regions associated with social interaction exhibit evidence for spontaneous mentalizing? (2) How does the neural similarity between social interaction and mentalizing change from middle‐childhood to early adolescence? (3) What are common neuro‐representational links between social interaction and mentalizing exhibited across social brain regions? To accomplish this, we utilized a larger sample of participants who underwent the experimental paradigm originally reported by Alkire et al. (2018), wherein participants played a guessing game with a live interaction partner who provided hints, as well as making guesses about an abstract character, the hints for which were generated by a computer. In both cases, half of the hints involved information about the target's mental state and half involved nonmental, physical information about the target to determine the right answer. This yielded a within‐subject, 2 (Peer/Character) × 2 (Mental/Nonmental) design that enabled us to quantify the similarity of brain activity patterns associated with offline mentalizing and social interaction, and assess fit with models about the underlying neurocognitive process involved (Figure 1).

We formalized three conceptual models of the neurocognitive links between social interaction and mentalizing. Consistent with the idea that social interaction engages spontaneous mentalizing, Model 1 (the “interaction engages mentalizing” model) proposes that interactions with a peer elicit a similar pattern of brain activity as when thinking about the mental state of a character, but that is dissimilar from the pattern of brain activity elicited by thinking about nonmental characteristics of a character. Alternatively, Model 2 (the “interaction” model) proposes that the interactive peer context and the character conditions elicit dissimilar patterns of brain activity from each other, but do not distinguish mentalizing and nonmental state reasoning from either target. Finally, Model 3 (the “*mentalizing*” model) proposes that mentalizing and nonmental state reasoning elicit dissimilar patterns of brain activity, but does not distinguish between peer and character conditions.

To address the first question of whether brain regions associated with social interaction show evidence for spontaneous mentalizing, we calculated the fit to each of our three conceptual models within a set of a priori regions of interest (ROIs) commonly implicated in studies of social interaction that were obtained from Neurosynth (Yarkoni et al., 2011). Additionally, convergent evidence for the ROI analysis was sought through exploratory whole‐brain searches for model fit using the searchlight approach (Supplemental Section 5). Because the transition to adolescence is marked by significant changes in social‐interactive contexts and mentalizing capabilities, we further assessed how neural similarity between social interaction and mentalizing changed from middle childhood to early adolescence by examining the relationship between age and model fit in each of the aforementioned social interaction ROIs. Finally, because we are interested in the contribution of brain structure associated with other social cognitive processes in linking social interaction and mentalizing, we conducted model‐free analyses in a larger set of “social brain” ROIs obtained from Alcalá‐López et al. (2018). The motivation for this set of analysis was to elucidate neuro‐representational links between social interaction and mentalizing that were not captured by our a priori models, and to uncover similarities across brain regions in terms of their neural similarity structure (also known as “representational connectivity”).

## 4 METHODS

### 4.1 Participants

A sample of 92 neurotypical 8‐ to 14‐year‐old participants were enrolled in a larger, multi‐session project investigating the neural correlates of social interaction during middle childhood. Participants were recruited from the greater Washington, DC area, and exclusionary criteria included MRI contraindications, diagnosis of neurological or psychiatric disorders, first‐degree relatives with autism or schizophrenia, and nonnative English speakers. A subset of 72 participants was selected for the current analyses because they completed at least two usable fMRI runs of the social interaction fMRI task described in Alkire et al. (2018) and believed in the manipulation that they were interacting with another peer in real time. Results from 28 participants of our sample of 72 are reported in Alkire et al. (2018), but using an orthogonal approach of group‐averaged, univariate activation. The minimum of two usable runs (i.e., 12 trials per condition) follows work indicating that neural representations can be captured with fewer trials than in traditional activation studies (Zeithamova et al., 2017), and follow‐up analyses using only participants with three and four usable runs were conducted and reported in the Supplemental Information for additional validation. A run was deemed usable if the average head motion during the run was under 0.5 millimeter (mm) framewise displacement as defined by Power et al. (2014), and if less than 10% of the volumes were censored (i.e., regressed out) for having movement over 1 mm framewise displacement. Of the final sample of 72 participants, 28 were female and 44 were male, with a mean (standard deviation) age of 10.8 (1.79), and an age range of 8–14.6 years (Table 1).

**TABLE 1:** Race/ethnicity information for the full sample

Race/ethnicity	Count (percent)
Asian	3 (4.2%)
Black/African American	26 (36.1%)
Hispanic/Latino	7 (9.7%)
Native American/Alaskan	2 (2.8%)
White	47 (65.3%)
Multiple races/ethnicities	10 (13.9%)
Prefer not to say/no response	1 (1.4%)

*Note*: Race and ethnicity categories are based on required National Institutes of Health reporting requirements and reflect the categories that participants were presented with as options, but are not necessarily aligned with current best practices for how race and ethnicity should be referenced.

### 4.2 Experimental protocol

The experimental protocol is the same as outlined by Alkire et al. (2018). Briefly, participants were instructed that they would be chatting with a peer in a different lab who would also be undergoing a brain scan, and were shown pictures of two age‐ and gender‐matched peers that they could choose from. In actuality, there were no live interaction peers, and every participant received the same stimuli. Participants were instructed on the “guessing game” that they played during the scan wherein participants were given a hint by their interaction partner on half the trials (Peer condition), and from the computer about a fictional story character for the other half of the trials (Character condition). It was the job of the participant to answer the question “Which will I/she/he pick?” (Mental condition) or “Which of these match?” (Nonmental condition) via button‐press to select the appropriate response from the two answer choices. Mental and Nonmental items were counterbalanced across participants such that each item was presented in the Peer and Character conditions roughly an equal number of times throughout data collection to prevent unintended biases. This yielded a fully within‐subject, 2 (Peer/Character) × 2 (Mental/Nonmental) design. The guess phase of each trial (encompassing the target cue, hint, and response options; Figure 1a) was modeled as our event of interest and was followed by a 2–6 s jittered period before feedback about the correct answer was provided for 2 s. The task was presented using PsychoPy (Peirce, 2007) over four functional runs, each with six trials per condition for a total of 24 trials per run. After scanning, participants completed a questionnaire asking about their enjoyment of and engagement with the Peer and Character conditions to assess the impact of the live‐interaction manipulation (additional details are provided in Supplemental Methods 1).

### 4.3 Behavioral data analysis

Analyses of behavioral task performance—accuracy (percent correct) and reaction time (RT) in seconds—and the postscan questionnaire were conducted using R (R Core Team, 2020) and JASP (JASP Team, 2020). The continuous variables of age, accuracy, and RT were first assessed using the Shapiro–Wilk test for normality, and data were transformed as needed to meet the assumptions of the parametric analysis of variance. Between‐group *t*‐tests for each condition's accuracy and RT were calculated to assess gender effects, and if either measure showed significant gender differences for any condition, gender was entered as a covariate in the subsequent analysis of variance for the measure. Correlations with age for each condition's accuracy and RT were calculated to assess age effects, and if either measure showed a significant correlation with age for any condition, age was entered as a covariate in subsequent analysis of variance for the measure. Accuracy and RT were each entered into two‐way repeated measures analysis of variance (with appropriate covariate as needed) to determine main effects of social interaction (Peer vs. Character) and mentalizing (Mental vs. Nonmental), and their interaction. Significant results were interrogated further with follow‐up *t*‐tests. Responses to the postscan questionnaire about participants’ enjoyment and attention were compared between the Peer and Character conditions using paired samples *t*‐tests.

### 4.4 MRI acquisition and data processing

FMRI data were acquired at the Maryland Neuroimaging Center on a 3.0 Tesla scanner with a 32‐channel head coil (MAGNETOM Trio Tim System, Siemens Medical Solutions). Four runs of the task were acquired using multiband‐accelerated echo‐planar imaging (66 interleaved axial slices, multiband factor = 6, voxel size = 2.19 × 2.19 × 2.20 mm, repetition time = 1250 ms, echo time = 39.4 ms, flip angle = 90°, pixel matrix = 96 × 96) followed by a structural scan (3D T1 magnetization‐prepared rapid gradient‐echo sequence, 192 contiguous sagittal slices, voxel size = 0.45 × 0.45 × 0.90 mm, repetition time = 1900 ms, echo time = 2.32 ms, flip angle = 9°, pixel matrix = 512 × 512), and opposite phase‐encoding fieldmap scans (66 interleaved axial slices, voxel size = 2.19 × 2.19 × 2.20 mm, repetition time = 7930 ms, echo time = 73 ms, flip angle = 90°, pixel matrix = 96 × 96). Neuroimaging data were preprocessed using fMRIPrep 1.4.1 (Esteban et al., 2019). Briefly, anatomical images were segmented and normalized to MNI space; functional images were skull‐stripped, susceptibility distortion corrected, realigned, slice‐time corrected, coregistered, and warped to the normalized anatomical image (see Supplemental Methods 2 for full fMRIprep preprocessing pipeline). Additionally, functional data were masked using subject/run‐specific masks generated by fMRIprep, and intensity normalized to a mean of 100 per voxel. Subject‐level, voxel‐wise multiple linear regression was calculated using AFNIs (Cox, 1996) 3dREMLfit for each run separately. The guess period, which includes the target cue (Peer/Character), hint, and responses options (Figure 1a), for each of the four conditions (i.e., Peer Mental, Peer Nonmental, Character Mental, and Character Nonmental) was modeled as our events of interest. This was achieved by convolving the guess period with the canonical hemodynamic response using a duration modulated response function (AFNIs dmBlock) with RT as duration to ensure that cognition related to the guess period was captured. The feedback period was modeled separately as events of no interest, along with the six motion parameters (x, y, z, roll, pitch, and yaw), their derivatives, and volumes censored due to framewise displacement >1 mm. This process yielded subject‐level *t*‐maps for each condition, which were used for all subsequent analyses.

### 4.5 Representational similarity analysis

**FIGURE 2:**
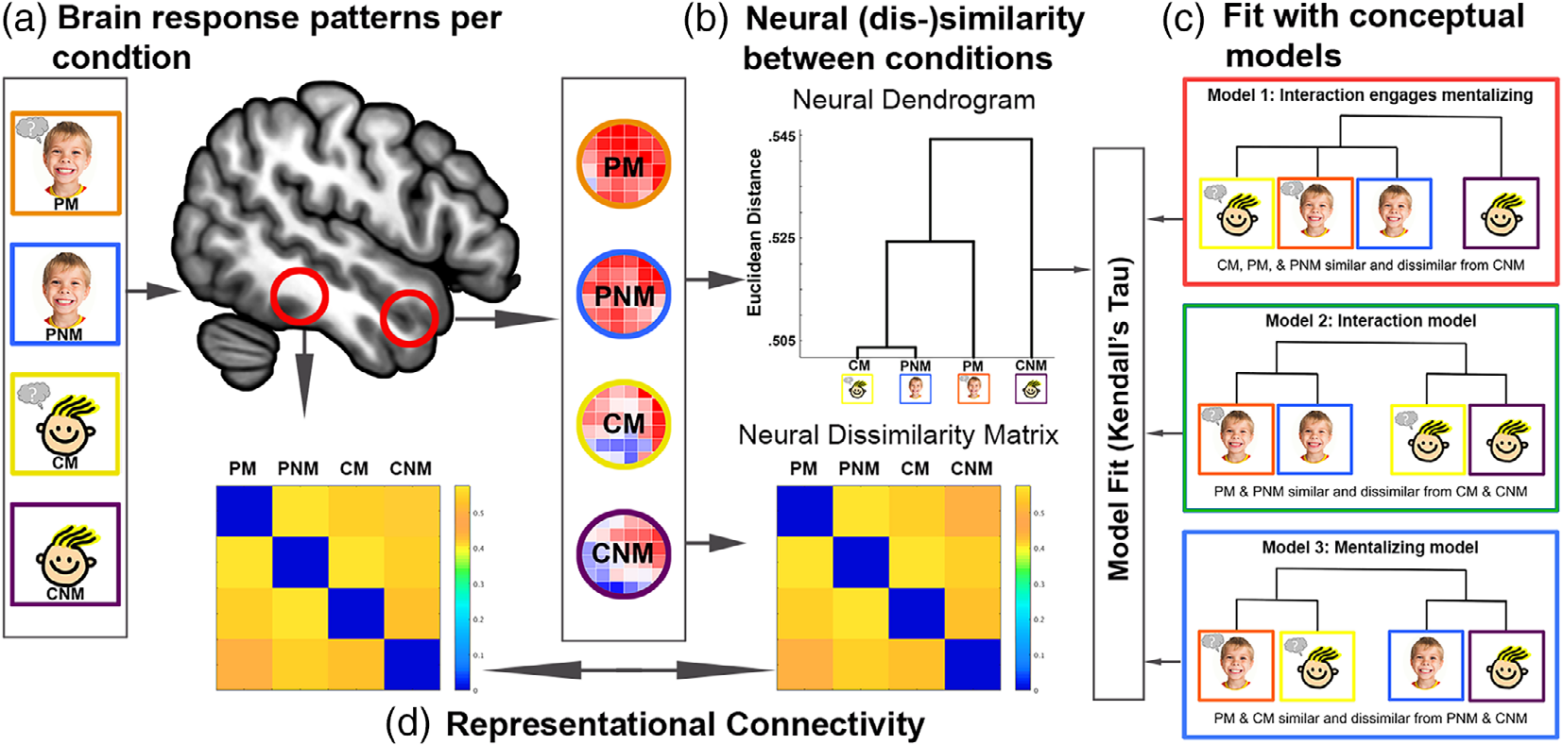
Steps for model‐based representational similarity analysis: We first estimate the voxel‐wise response pattern for each condition for a given brain region using unsmoothed subject‐level models (a), then calculate the Euclidean distance between response patterns for each pair of conditions to construct our neural dissimilarity matrices (which can also be visualized as dendrograms; b), and estimate fit to each of our models by calculating Kendall's tau‐a (c). For the representational connectivity analyses, you examine the fit between the neural dissimilarities between brain regions (d)

Neural representational dissimilarity matrices (RDMs) were calculated per subject for each ROI by extracting voxel‐wise *t*‐values associated with each condition, using the voxel‐wise values to calculate the Euclidean distance between each pair of conditions using the CoSMoMVPA toolbox for MATLAB (Oosterhof et al., 2016), and normalizing the RDMs by subtracting the minimum Euclidean distance and dividing by the range. Neural RDMs were then tested against three conceptual models of hypothesized relationships between conditions, which were formalized as binary RDMs wherein 0 = similar and 1 = dissimilar. Model 1, or the “*interaction engages mentalizing*” model, proposes that all interactions with a peer, regardless of explicit mental state information, elicit a similar pattern of brain activity as thinking about the mental state of a character, which is dissimilar from the pattern of brain activity elicited when thinking about nonmental characteristics of a character. That is, this model formalizes Character Mental and both Peer conditions as being similar to each other (i.e., condition‐pairs between each are 0's), and each are dissimilar (i.e., have 1's) from Character Nonmental condition. Model 2, or the “interaction” model, states that both Peer conditions are similar to each other and both Character conditions are similar to each other, but Peer and Character conditions are dissimilar from each other. Model 3, or the “mentalizing” model, states that both Mental conditions are similar to each other and both Nonmental conditions are similar to each other, but Mental and Nonmental conditions are dissimilar from each other (Figure 2).

Model fit was estimated by calculating Kendall's Tau‐a rank‐order correlation coefficient between the off‐diagonal elements of the neural and model RDMs using the RSA toolbox for MATLAB (Nili et al., 2014). For each ROI, model fit was calculated between each participant's neural RDM and the three model RDMs, and were transformed to *z* value using the Kendall's Tau normal approximation formula (*z* = 3τ*√*n*(*n* − 1)/√2(2*n* + 5)). The model fit estimates were entered into a Bayesian multilevel (BML) model using AFNIs RBA program (Chen et al., 2019) to assess the strength of evidence in favor of each model per ROI. This approach confers multiple advantages over the traditional null hypothesis significance testing framework—the ability to get around the issue of multiple comparison corrections through the calculation of a single model that accounts for the hierarchical structure of the data, better model fit afforded by the estimation of informed priors using partial pooling of data across levels, and the full reporting of results rather than dichotomizing findings based on significance thresholds (Chen et al., 2021). As a proof‐of‐concept, we used the aforementioned approach to conduct a preliminary re‐analysis of the brain regions reported by Alkire et al. (2018) that exhibited overlapping univariate activations for social interaction and mentalizing, indicative of Model 1 fit. RSA revealed that only two of the four ROIs exhibited strong evidence for Model 1 fit (the “interaction engages mentalizing” model), which demonstrated the sensitivity of RSA and motivated the subsequent analyses (Figure S1). The code for all RSA is available at https://github.com/JunaidMerchant/CMNT_RSA.

### 4.6 Model fit analysis of social interaction ROIs

We conducted a set of theory‐driven analyses utilizing a set of ROIs obtained through NeuroSynth (Yarkoni et al., 2011) to examine representational similarity within brain regions associated with social interaction. The motivation behind this set of analyses was to examine brain areas associated with social interaction that may exhibit patterns of activity indicative of mentalizing, thereby providing a better understanding of the role of mentalizing in interactive contexts. To this end, we first binarized (threshold = 0.5) and combined (addition) the meta‐analytic maps from NeuroSynth association tests of the search terms “social interaction” and “social interactions” using AFNIs 3dcalc. The resulting map was thresholded to a minimum cluster size of 50 voxels (nearest neighbor = 3) to retain ROIs of theoretical importance while eliminating numerous small clusters. This resulted in a set of 13 ROIs: dmPFC, ventromedial PFC (vmPFC), perigenual anterior cingulate cortex (pgACC), subgenual ACC (sgACC), bilateral TPJ, bilateral ATL, bilateral cerebellum (Crblm), left caudate, right inferior temporal gyrus (ITG), and right ventrolateral PFC (vlPFC). Model fit estimates were evaluated for each ROI using the BML procedure described in the previous section. To aid the reader in the interpretation of these results, we focus on regions showing “very strong,” “strong,” and “moderate” evidence of positive model fit as indicated by the intercept falling beyond 97.5, 95%–97.5%, and 90%–95% quantile intervals, respectively, under BML. These values were chosen because they correspond with a two‐tailed *p* values of .025, .05, and .1, respectively, under conventional statistical testing framework (e.g., Xiao et al., 2019). Of note, the focus on model fit in the positive direction is because positive model fit has a meaningful interpretation. Age related effects on model fit were evaluated by calculating the rank‐order correlations between age and model fit estimates only for the ROIs exhibiting moderate to very strong evidence in favor of a model fit.

### 4.7 Model‐free analysis of the social brain ROIs

Model‐free analyses were conducted to evaluate if other, nonhypothesized relations between task conditions exist in the social brain, and to examine the organization of these regions based on pattern similarity structure. That is, rather than examining fit to prespecified models, “representational connectivity” analysis estimates the relationship between brain areas in terms of how similar they represent the four conditions. This allows us to uncover clusters of brain regions that are “representationally connected” in terms of how they link social‐interactive and mentalizing processes. To this end, we started with a set of 36 publicly available ROIs derived from an extensive set of meta‐analytic, data‐driven analyses of 3972 social neuroscience studies using fMRI and/or PET imaging (Alcalá‐López et al., 2018). The ROIs were resampled to the resolution of our functional data and inflated within a gray matter mask informed by white matter and CSF skeleton to maintain a comparable amount of brain coverage and attention to anatomical contours. The resulting ROIs had a uniform volume of 120 voxels.

Analyses of these social brain ROIs proceeded in three major steps. Step 1: because the social brain ROIs cut across many different brain systems that may not be involved in any of our task conditions, it was necessary to calculate the lower bound of the “noise ceiling,” which expresses how consistent the representations are across participants. Noise ceiling calculation was conducted for each ROI and tested against zero (i.e., no consistency across participants) to determine their inclusion in further analyses (Lage‐Castellanos et al., 2019). Noise ceiling was calculated by creating an average neural RDM across all but one participant, calculating Kendall's Tau‐a between this average RDM and the neural RDM from the left‐out participant, and iterating this process across participants. The Kendall's Tau‐a coefficients were then tested against zero using Wilcoxon signed‐rank tests, and ROIs with a noise ceiling significantly above chance were retained.

Step 2: the resulting ROIs were interrogated using exploratory factor analysis (EFA; Fabrigar & Wegener, 2011) to determine the factor structure of the response patterns across the ROIs, which elucidated the number of groups that the ROIs were separated into based on representational connectivity (i.e., shared similarity structures between ROIs). This was conducted by first averaging the neural RDMs across participants for each ROI, and entering the averaged Euclidean distances into an EFA using minimum residual extraction and oblimin rotation (though results from principal axis extraction and other rotation methods yielded similar results). A cut‐off eigenvalue of 1 and a minimum of 80% explained variance were used to determine the factor solution. Step 3: the Euclidean distances were used to calculate correlations between each pair of ROIs, which were submitted to a hierarchical cluster analysis using Ward's method (Ward, 1963) to identify clusters of regions with similar neural similarity structures, and the number of clusters were set to the number of factors determined from the EFA. The neural RDMs for each ROI within each cluster were averaged together and qualitatively examined for a better understanding of the neural representations of each cluster of ROIs.

## 5 RESULTS

### 5.1 Behavioral data analysis

Shapiro–Wilk tests for normality revealed that accuracy measures violated the assumption of normality (Table S3). Arcsin square root transformation of accuracy scores (the recommended transformation for percentages) did not fix the distribution of these measures to pass the Shapiro–Wilk tests for normality (nor any other transformations). Because there are no nonparametric equivalents of the two‐way repeated‐measures ANOVA, we proceeded with the parametric analyses as planned, but reported results of nonparametric tests for each of the significant effects we obtained from the accuracy ANOVA (Table S6).

No significant gender effects were revealed from the independent samples *t*‐tests of the accuracy and RT scores for each simple condition, and when using nonparametric tests (all *p* values >.16), thus gender was not included in the analyses of variance reported below (however, ANCOVAs with gender as a covariate can be found in the Supplemental document and are consistent with the results reported below). There were significant negative correlations with age for all RT measures (all *p* values <.004), indicating that participants responded more quickly with age. Using Spearman's rank‐order correlation, significant positive correlations with age were revealed for accuracy on all Mental trials (combining Character Mental and Peer Mental), *rho*(70) = .28, *p* = .02, and Character Mental trials, *rho*(70) = .25, *p* = .017, indicating better performance on these trials with increasing age. Age was therefore subsequently used as a covariate for the accuracy and RT analyses of variance.

**FIGURE 3:**
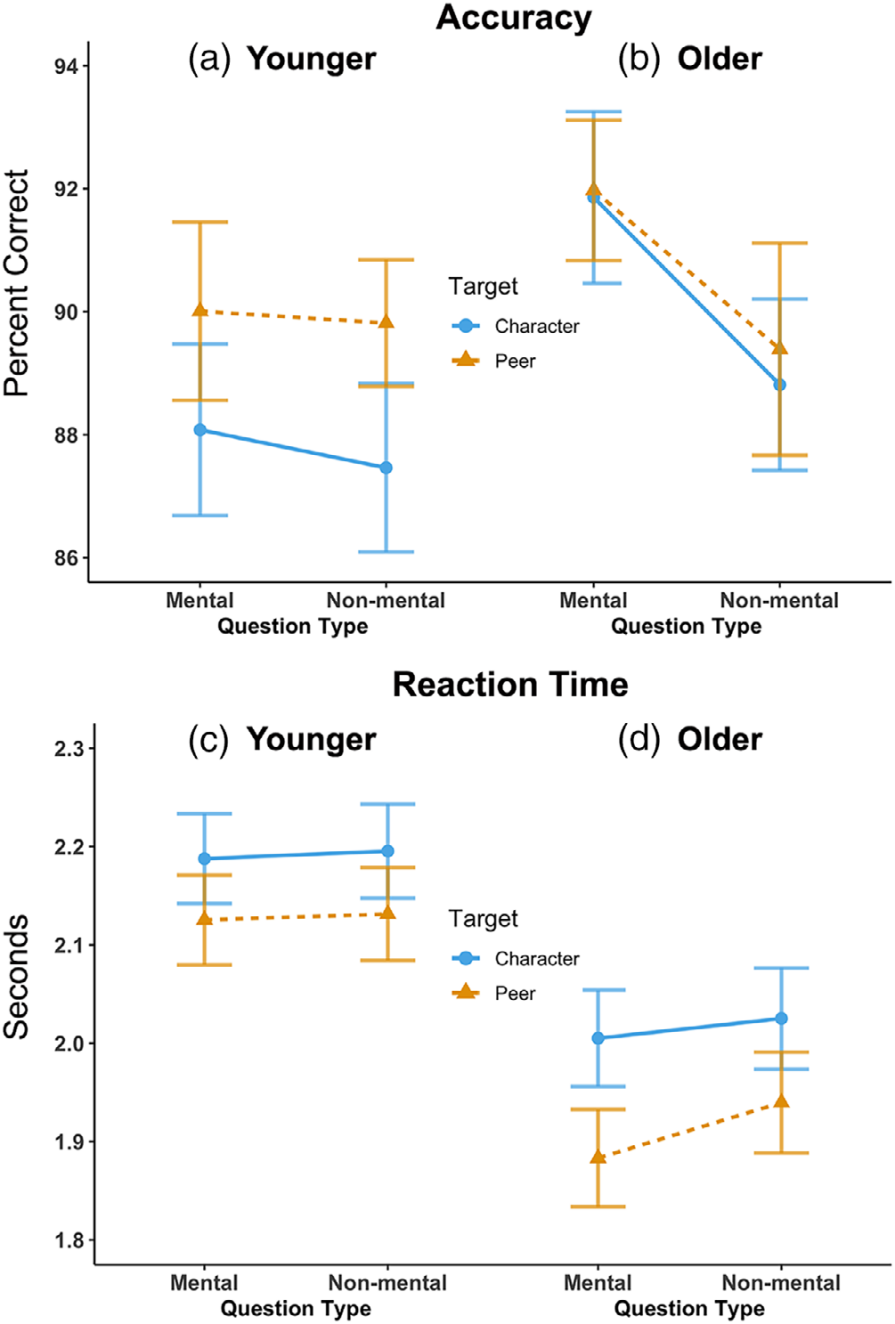
Visualizations of the 2 × 2 analysis of covariance for accuracy (a) and reaction time (b). Plots use median split on age to visualize the interactions even though the analyses were conducted using age as a continuous variable

A 2 (Target: Peer vs. Character) × 2 (Question type: Mental vs. Nonmental) repeated measures ANCOVA with age as a covariate on accuracy scores revealed a main effect for question type, *F*(1,70) = 4.83, *p* = .031, indicating higher accuracy for Mental questions, and an age‐by‐question type interaction, *F*(1,70) = 6.99, *p* = .01, such that the difference in accuracy between Mental and Nonmental questions is greater with increasing age (Figure 3a; Table 2a). The same pattern of results was obtained when including gender as an additional covariate (Table S5), and were partially validated by nonparametric tests (Table S6). A 2 (Target: Peer vs. Character) × 2 (Question type: Mental vs. Nonmental) repeated measures ANCOVA with age as a covariate on RT scores revealed a main effect for age, *F*(1,70) = 14.24, *p* < .0005, indicating faster overall RT with age; a significant target‐by‐question type interaction, *F*(1,70) = 4.19, *p* = .044, such that there is a bigger RT difference between Mental and Nonmental for the Peer condition; and a significant three‐way interaction, *F*(1,70) = 4.78, *p* = .032, such that the difference between Peer Mental and Peer Nonmental gets bigger with age (Figure 3b; Table 2b). A similar pattern of results was obtained when including gender as an additional covariate (Table S4).

**TABLE 2:** Full report of (a) accuracy and (b) reaction time ANCOVAs

(a) Accuracy ANCOVA						
Within subjects effects	Sum of squares	df	Mean square	*F*	*p*	*η* ^2^
Target	0.013	1	0.013	3.732	.057	7.00E−03
Target × Age	0.01	1	0.01	2.782	.1	0.005
Residuals	0.251	70	0.004			
Question type	0.014	1	0.014	4.834	.031	7.00E−03
Question type × Age	0.02	1	0.02	6.994	.01	1.00E−02
Residuals	0.205	70	0.003			
Target × Question type	0.004	1	0.004	1.421	.237	0.002
Target × Question type × Age	0.004	1	0.004	1.322	.254	0.002
Residuals	0.197	70	0.003			

Between subjects effects					
Age	0.029	1	0.029	1.682	0.199
Residuals	1.228	70	0.018		

Descriptives				
Target	Question type	Mean	SD	N
Character	Mental	0.9	0.085	72
	Nonmental	0.881	0.083	72
Peer	Mental	0.91	0.078	72
	Nonmental	0.896	0.085	72

(b) Reaction time ANCOVA						
Within subjects effects	Sum of squares	df	Mean square	*F*	*p*	η^2^
Target	0.008	1	0.008	0.673	.415	2.95E−04
Target × Age	0.043	1	0.043	3.655	.06	0.002
Residuals	0.815	70	0.012			
Question type	0.014	1	0.014	1.358	.248	5.42E−04
Question type × Age	0.023	1	0.023	2.21	.142	8.82E−04
Residuals	0.744	70	0.011			
Target × Question type	0.05	1	0.05	4.194	.044	0.002
Target × Question type × Age	0.057	1	0.057	4.779	.032	0.002
Residuals	0.84	70	0.012			

Between subjects effects					
Age	4.057	1	4.057	14.235	3.34E−04
Residuals	19.952	70	0.285		

Descriptives				
Target	Question type	Mean	SD	N
Character	Mental	2.096	0.297	72
	Nonmental	2.11	0.308	72
Peer	Mental	2.004	0.309	72
	Nonmental	2.036	0.309	72

*Note*: Type III sum of squares.

Paired‐sample *t*‐tests of the postscan questionnaire replicated the results reported in Alkire et al. (2018), such that participants reported greater enjoyment and paid more attention during the Peer compared to the Character conditions, all *p*s < .001. Exploratorily, we compared self‐reported difficulty when making guesses about the Peer versus Character, which indicated that participants found the conditions equally challenging, *p* = 0.65 (Table S7).

### 5.2 Social interaction ROIs

#### 5.2.1 Model fit analyses

**FIGURE 4:**
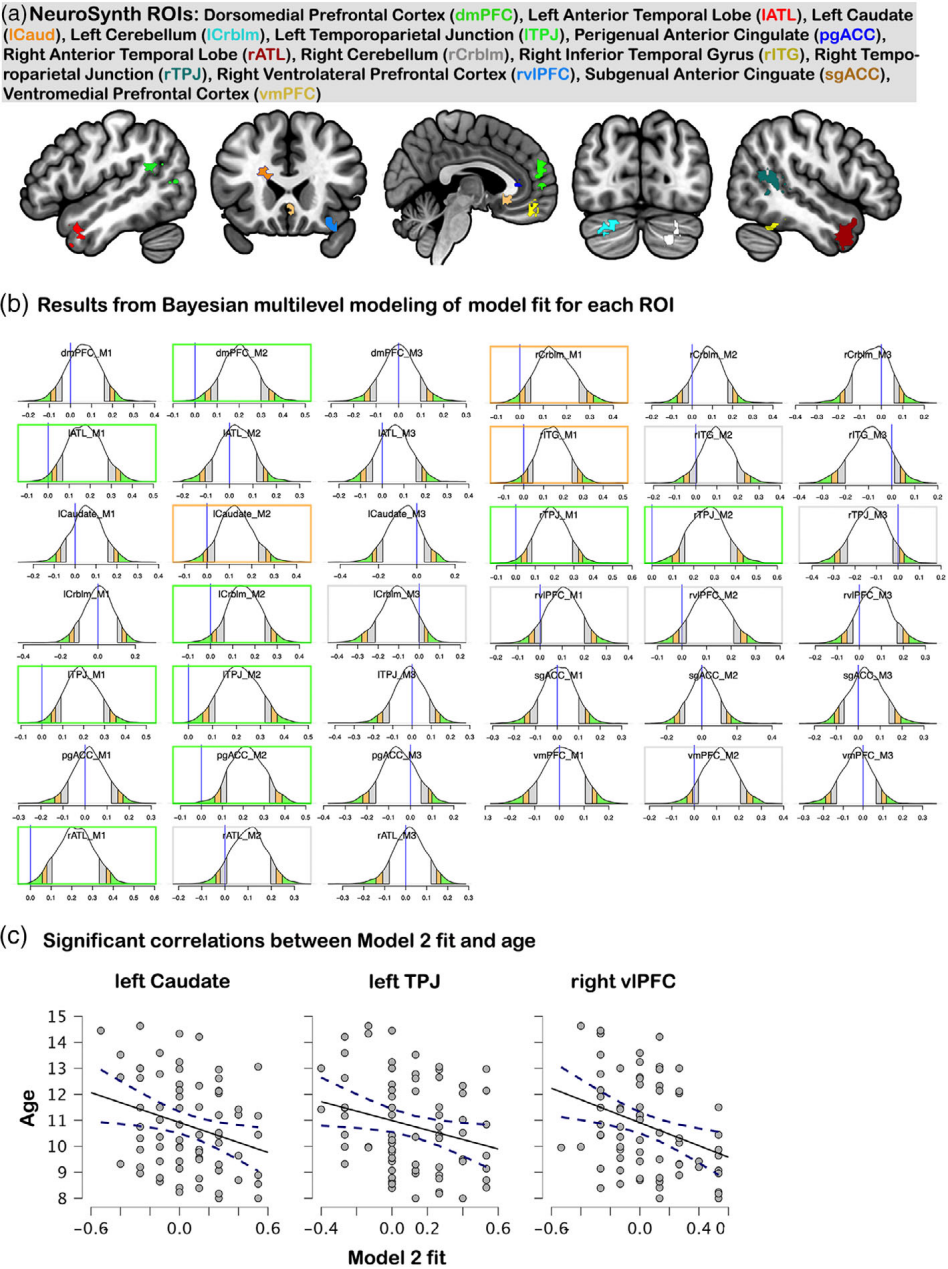
(a) Social interaction ROIs obtained from Neurosynth, (b) plots from Bayesian multilevel models on model fit for each ROI (b), and (c) scatter plots visualizing the correlation of model 2 fit and age for the left caudate, left TPJ, and right vlPFC. Colors in the BML graph indicate which quantile level each ROI falls in, with green = 97.5% quantile or more (very strong evidence), orange = 95%–97.5% quantile range (strong evidence), gray = 90%–95% quantile range (moderate evidence)

Across the 13 social interaction ROIs, four exhibited very strong evidence in favor of Model 1 fit in our sample: bilateral ATL and bilateral TPJ as indicated by the intercept falling beyond the 97.5% quantile of the ROIs' posterior distributions. Model 1 fit in the bilateral ATL converges with findings from the re‐analysis of the conjunction ROIs which also revealed moderate to strong evidence for Model 1 fit in these regions (Supplemental Section 2). Additionally, the right cerebellum and right ITG showed strong evidence in favor of Model 1 fit as indicated by the intercept falling in between the 95% and 97.5% quantiles of the ROIs' posterior distribution, and the right vlPFC exhibited moderate evidence for Model 1 fit as indicated by the intercept falling in between the 90% and 95% quantiles of the ROIs posterior distribution. Five ROIs also exhibited very strong evidence in favor of Model 2 fit in our sample (bilateral TPJ, dmPFC, left cerebellum, and perigenual ACC), one ROI exhibited strong evidence in favor of Model 2 fit (left caudate), and four ROIs exhibited moderate evidence in favor of Model 2 fit (right ATL, right ITG, right vlPFC, and vmPFC; Figure 4b). Only two ROIs exhibited moderate evidence supporting Model 3 fit (left cerebellum and right TPJ). Follow‐up paired‐sample Wilcoxon signed‐rank tests on the fit estimates for Models 1 and 2 for bilateral TPJ, right ITG, and right vlPFC (the ROIs showing evidence for fit in both models) indicated no significant differences in the model fit estimate (*p* values = .44–.96), suggesting that the similarity in voxel‐wise patterns between conditions in these regions support multiple types of representations.

Additional support for the Model 1 fit in the left ATL comes from our supplemental searchlight analyses that revealed clusters in the left ATL and right posterior superior temporal sulcus (pSTS; Supplemental Section 5), however, these were uncovered using an exploratory uncorrected threshold, so interpretations must be made with caution. The searchlight analyses provided additional support for Model 2 fit in the dmPFC and bilateral TPJ, which also uncovered clusters in the visual cortex, left pSTS, left IFG, and frontal pole (Figure S4b and Table S10b). Searchlight for Model 3 did not reveal a significant fit in any region, even at exploratory threshold values.

### 5.3 Age‐related differences in model fits

Of the 17 ROI × model fit estimates exhibiting moderate to very strong evidence in favor of model fit, three had significant negative correlations between age and Model 2 fit when assessed using Spearman's rank‐order correlation (no significant correlations with Model 1 fit): the left caudate, *rho*(70) = −.254, *p* = .031, left TPJ, *rho*(70) = −.243, *p* = .04, and the right vlPFC, *rho*(70) = −.308, *p* = .008. However, false discovery rate correction for 17 correlations calculated rendered no age × model fit correlation significant, thus these results should be interpreted with caution (Figure 4c). Nonetheless, follow‐up correlations and regression analyses were calculated using the between‐condition Euclidean distances for each of these ROIs to gain a better understanding of what might be driving the decreasing model fit. This revealed a significant positive correlation between age and Euclidean distance between Peer Mental and Peer Nonmental conditions in the left caudate, *rho*(70) = −.254, *p* = .031, which was confirmed by regression analyses that controlled for the effect of the other between‐condition distances. No significant correlations with age were found for the between‐condition distances in the left TPJ. The right vlPFC exhibited a significant negative correlation between age and the distance between Character Mental and Peer Nonmental conditions, *rho*(70) = −.259, *p* = .028, and a significant positive correlation between age and the distance between Peer Mental and Peer Nonmental conditions, *rho*(70) = .234, *p* = .048, which were partially confirmed by regression analyses that controlled for the effect of the other between‐condition distances (Table S9). The finding that both the caudate and right vlPFC exhibited a positive correlation between age and distance between the Peer Mental and Peer Nonmental conditions suggests that as children transition to adolescence, they develop increasingly distinct representations of different types of information they process about their interaction partner.

### 5.4 Model‐free analyses within the social brain

Analyses to examine the similarity structure of the social brain ROIs proceeded in three steps: (1) ROI selection based on significantly greater than zero noise ceiling; (2) exploratory factor analysis to determine the number of factors underlying the similarity structures of the selected ROIs; (3) hierarchical clustering and qualitative examination of the similarity structures of the ROIs clustered together.

**FIGURE 5:**
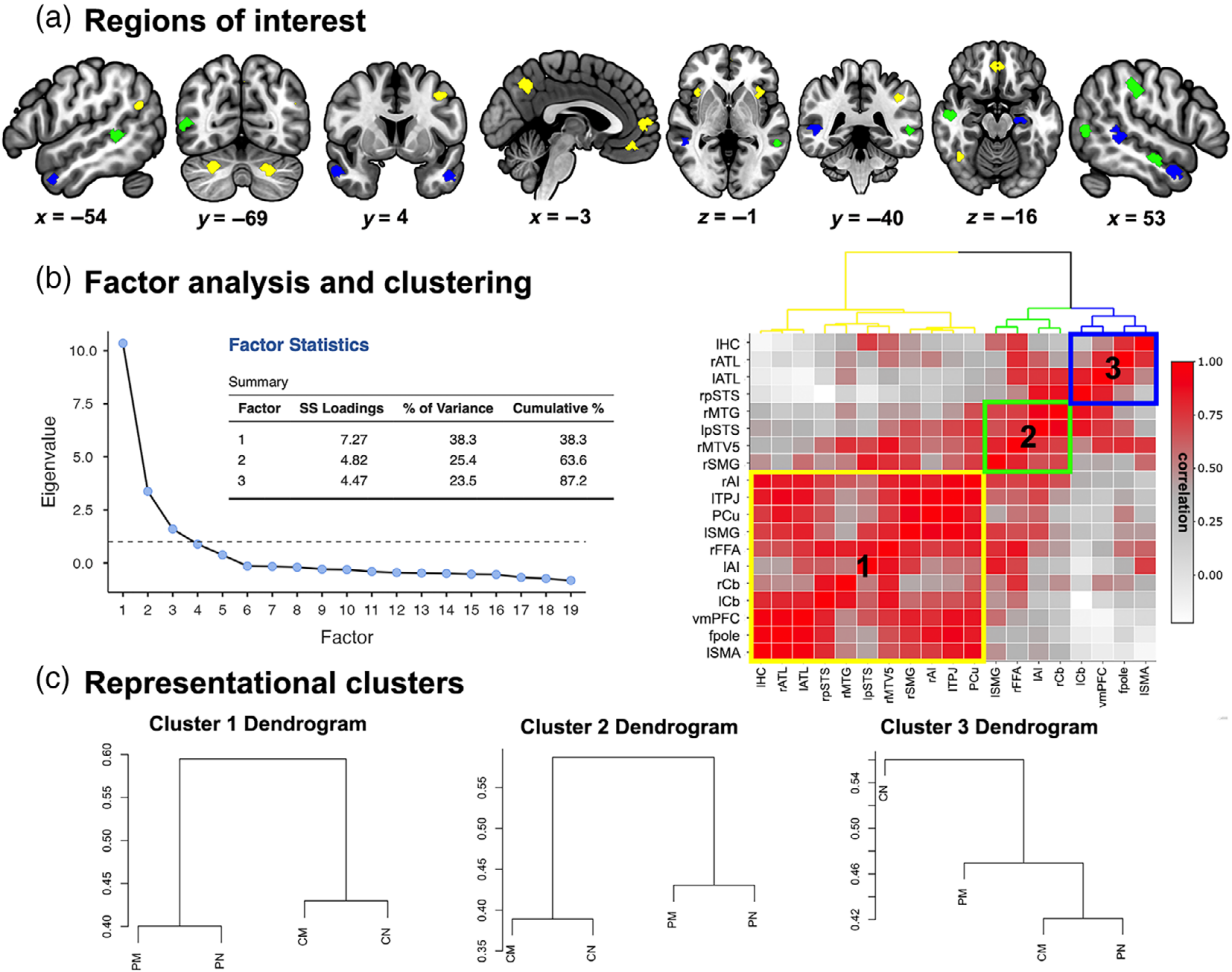
Social brain regions of interest (ROI) used in the representational connectivity analysis (a), and the results from the exploratory factor analysis and hierarchical clustering (b). Dendrograms of neural dissimilarity for each cluster averaged across the ROIs comprising the cluster (c)

Noise ceiling calculation revealed that 19 of the 36 ROIs were significantly above chance, suggesting that these ROIs contain consistent representations across our sample. This included bilateral ATL, bilateral pSTS, bilateral supramarginal gyrus (SMG), bilateral cerebellum (Cb), bilateral AI, left supplementary motor area (SMA), frontal pole (fpole), vmPFC, precuneus (PCu), left hippocampus (HC), left TPJ, right middle temporal gyrus (MTG), right fusiform face area (FFA), and right middle temporal V5 area (MTV5). Thus, these 19 were included in the next step of exploratory factor analysis (Figure 5a).

Exploratory factor analysis of these 16 ROIs yielded a 3‐factor solution that explained 87.2% of the variance in Euclidean distances. Hierarchical clustering using Ward's method was used to estimate three clusters that minimized the total within‐cluster variance (Figure 5b).

Qualitative examination of the averaged Euclidean distances of each cluster of ROIs revealed that Cluster 3 (bilateral ATL, right pSTS, and left HC; in blue) resembled Model 1, in that PM, CM, and PNM were similar to each other, and different from CNM. Clusters 1 and 2 exhibited a pattern similar to Model 2, but differed in how well they distinguished the Peer and Character conditions. Averaging the similarity structures of the ROIs comprising Cluster 1 (vmPFC, frontal pole, left SMA, bilateral AI, left SMG, PCu, left TPJ, right FFA, and bilateral Cb; in yellow) revealed that, while the greatest dissimilarities were between the Peer and Character conditions, the two Character conditions were relatively more dissimilar from each other than the two Peer conditions. The ROIs comprising Cluster 2 (left pSTS, right MTG, right SMG, and right MTV5; in green) showed the reverse pattern, such that, while the greatest dissimilarities were again between the Peer and Character conditions, the two Peer conditions were relatively more dissimilar from each other than the two Character conditions (Figure 5c). Together, qualitative assessment of Clusters 1 and 2 suggest that regions exhibiting Model 2 fit might have differing sensitivities to either the Peer or Character conditions.

## 6 DISCUSSION

This is the first study to evaluate the assumption that mentalizing is spontaneously engaged during social interactions by assessing the similarity in brain response patterns associated with mentalizing and peer interactions. Across our analyses we found the most consistent evidence that offline mentalizing and live peer interactions elicit similar patterns of brain activity in the left ATL, but a dissimilar pattern of activation when making nonmental state inferences about an abstract character (i.e., the “interaction engages mentalizing” model or Model 1). Our findings align with traditional, offline studies of mentalizing that have demonstrated differences in brain activity in this region when making mental versus nonmental inferences about abstract others (Aichhorn et al., 2009; Andrews‐Hanna et al., 2014; Dodell‐Feder et al., 2011), and add to the literature by demonstrating that patterns similar to mentalizing are elicited during social interactions that do not explicitly require mental state reasoning. In addition to providing support for the idea that mentalizing is spontaneously engaged in social‐interactive contexts, our results suggest that the ATL and other temporal regions may contribute to the integration of online and offline social‐cognitive processes. That is, social interactions involve the dynamic interplay between social knowledge that is available offline, such as an understanding of mental state concepts, and the online demands of reciprocal social exchange. This interpretation also fits with recent reviews describing how we get to know others, which involves acquiring and updating person‐specific knowledge through online engagements with them, and implicates the ATL and pSTS in this process (Anzellotti & Young, 2020; Kovács, 2020).

Consistent with the idea that the ATL supports the integration of online and offline social processes, different lines of research have demonstrated the convergence of multimodal, social information processing streams in the anterior portions of the temporal lobe. Ventral areas of the ATL have been associated with the representations of person‐specific knowledge (e.g., names, faces, and biographical information; Anzellotti, 2017; Blank et al., 2015; Borghesani et al., 2019; Kriegeskorte et al., 2007; Wang et al., 2017), while more dorsal, anterior portions of the ATL are considered to be a semantic hub for abstract social concepts (including mental‐state concepts; Arioli et al., 2020; Olson et al., 2007, 2013; Skipper et al., 2011; Wang et al., 2019; Zahn et al., 2007). Conceptually, our findings span the middle ground between the aforementioned lines of research in that our results suggest that peer‐specific and abstract knowledge about mental states are integrated and represented similarly in the ATL. This interpretation is supported by research indicating that the interplay between ventral and dorsal ATL contributes to the successful encoding and retrieval of information associated with a person's identity (Perrodin et al., 2015; Rice et al., 2018; Tsukiura et al., 2010). Moreover, this work has demonstrated left lateralization for associating person‐specific information with the individual's name (Abel et al., 2015; Borghesani et al., 2019; Olson et al., 2013; Tsukiura et al., 2008), which provides some explanation as to why our most consistent findings were in the left ATL, since our stimuli used proper names. However, even though our findings are consistent with the different functional accounts of the ATL mentioned above, additional work is needed to disentangle the specific computations engaged by the ATL that links social interaction and mentalizing.

Many of the other brain regions we examined exhibited fit to our “interaction” model that distinguishes the Peer and Character conditions (Model 2), but does not distinguish between Mental and Nonmental state reasoning for either target. This included brain regions of the mentalizing network (TPJ and dmPFC), as well as regions of the frontoparietal network (IFG, and IPL), the reward and value systems (caudate and vmPFC), and large swaths of the visual cortex (Supplemental Section 5). Model 2 fit in the visual cortex highlights the sensitivity of RSA to lower‐level visual dis/similarities between the stimuli used in Peer and Character conditions (i.e., differences in color and shape of the hints), while Model 2 fit in other regions likely reflects key differences in attentional, motivational, and social cognitive demands between real‐time engagement with a live social partner and offline assessments of an unknown character. Model‐free examination of the social brain ROIs provided additional support for this interpretation, in that the majority of the social brain exhibited similar structures that clearly distinguished between the Peer and Character conditions (i.e., Figure 5c). Representational connectivity further revealed two variations of the similarity structure that would fall under our Model 2, suggesting subtle differences in the underlying neurocognitive representations that are sensitive to either the Peer or Character conditions. Together, our findings add nuance to the assumption of second‐person neuroscience that social cognition during social interaction is fundamentally different from what has been revealed using third‐person approaches (Redcay & Schilbach, 2019; Schilbach et al., 2013) by demonstrating differing sensitivities to second‐ and third‐person contexts across brain regions, but confirmatory research is needed to provide support for this interpretation.

Our findings are also consistent with previous work demonstrating changes in both mentalizing and social interaction as individuals transition from middle childhood to adolescence (Richardson et al., 2020). Accuracy scores indicate that mentalizing ability improves with age (i.e., age‐by‐question‐type interaction), and reaction time measures suggest that this might be driven by an increased efficiency in making mental state inferences specifically for peers (i.e., significant three‐way interaction between age, question type, and target; Figure 3 and Table 2). These behavioral effects align with age‐related decreases in fit with the “interaction” model (Model 2) in the left caudate and right vlPFC, which was driven by increasing neural dissimilarity between the Peer Mental and Peer Nonmental conditions. Because the vlPFC and caudate are associated with reward and valuation (Bartra et al., 2013; Nejati et al., 2018), this result may relate to the ever‐increasing importance of peers, and particularly the need to understand their perspectives, during this age range. Indeed, research has demonstrated heightened sensitivity of the reward system to social contexts during the transition into adolescence (Chein et al., 2011; Moreira & Telzer, 2018; Smith et al., 2015), and a related increase in the tendency to automatically integrate peer perspectives into self‐evaluations (Jankowski et al., 2014; Pfeifer et al., 2009; Van der Cruijsen et al., 2019). It is worth noting that the conditioned social reward associated with “texting” might confound the inherent reward experienced in social interactive contexts since the Peer condition stimuli were presented in a format that resembles text messages, especially given that the age range of our sample is when individuals start obtaining their own mobile devices to chat with their peers. However, this interpretation is hard to reconcile with the fact that the age‐related changes we found were for increasing dissimilarity between the two Peer conditions, which would not be expected if the reward‐related activity was driven by associations with texting in general. Nonetheless, the current findings add to our understanding of the development of social cognition by demonstrating that improvements in mentalizing capacity (or propensity to do so) during the transition to adolescence are particularly salient in social‐interactive contexts.

The developmental findings of the current project were enabled, in part, by the rapid changes in social and neural development that occur during the pre‐adolescent to early adolescent ages (Kilford et al., 2016; Mills et al., 2014), but our snapshot of this narrow age range also limits the generalizability of our results to other ages and populations. Thus, it is unknown whether the age‐related differences in the representational similarity structures we observed would continue in a linear trajectory as individuals transition into late adolescence and adulthood, or if they would return to pre‐adolescent levels similar to the inverted‐U shape trajectories observed in other social‐cognitive domains (Kilford et al., 2016; Somerville et al., 2013). This unknown is particularly challenging for many of our results in cortical regions associated with social cognition that undergo substantial structural changes well into adulthood, even though whole‐brain volume remains relatively stable after late childhood (Mills et al., 2014, 2016). At a theoretical level, although much of the motivation of the current work was to understand if mentalizing is a constituent process of social interaction, others have argued for the primacy of early social‐interactive experiences as the driving force for later‐developing social‐cognitive abilities, like mentalizing outside of social‐interactive contexts (Schilbach, 2014). That is, early social interactions may enable the neural architecture upon which offline mentalizing abilities develop. Together, our findings add to the developmental social cognitive literature, but data from a sample with a wider age range is needed to fill in the developmental trajectory of the relationship between offline mentalizing and social interaction.

In addition to advancing our theoretical understanding of the development of social cognition, the current work also demonstrates the utility of pattern‐based analyses for uncovering links between constituent processes involved in social behavior. The re‐analysis of the brain regions reported by Alkire and colleagues that showed overlapping activations for social interaction (without explicit mentalizing demands) and offline mentalizing (Supplementary Section 2) provided proof‐of‐concept for the sensitivity of RSA to elucidate underlying neurocognitive relationships that were unobtainable through univariate analyses. Despite expectations that overlapping activations were indicative of the “interaction engages mentalizing” model (Model 1), only two of these regions exhibited robust evidence for Model 1 fit, and two exhibited very strong evidence in favor of the “interaction” model that distinguishes the Peer and Character conditions (Model 2; Supplemental Section 2). However, our approach had limitations. First, because our models are not completely orthogonal, some ROIs exhibited comparably strong evidence for fit to Models 1 and 2 that were not disambiguated by direct comparison of model fit estimates (e.g., bilateral TPJ from social interaction ROIs; Figure 4b). Thus, it is unclear whether these regions might be subserving multiple functions or if this is merely an artifact of the way our models are set up. This limitation in modeling approach reflects our factorial task design, which also presents the limitation of dichotomizing the Peer and Character conditions. That is, research has demonstrated that responses in the mentalizing network are sensitive to factors like target familiarity and closeness (Laurita et al., 2017; Tacikowski et al., 2013) and how human‐like the interacting agent appears (i.e., computers versus anthropomorphic robots versus human; Krach et al., 2008; Takahashi et al., 2014). Thus, it is possible that growing familiarity with the Peer during the course of the experiment and differences in how realistic the Character was perceived to be may have muddied our ability to distinguish between fit to the different models.

Potentially related to the limitations of our modeling approach, it is noteworthy that we found only moderate evidence for two regions (left cerebellum and right TPJ) exhibiting fit to the “mentalizing” model that distinguishes the Mental and Nonmental conditions (Model 3), especially since previous work in our lab using the same experiment revealed activation differences between the Mental and Nonmental conditions in many regions. Model 3 fit in the right TPJ is supported by multi‐voxel investigations of mentalizing fMRI data that demonstrate decodable mental state representations in this region (Koster‐Hale et al., 2013; Koster‐Hale et al., 2017; Richardson et al., 2020; Weaverdyck et al., 2021), but we did not find convergent evidence for this finding through our searchlight analysis. There are several reasons as to why this might be the case. First, as in previous studies (e.g., Gweon et al., 2012), our task tightly controls for social information processing by matching conditions with equally social stimuli that do not include mental‐state information (i.e., the Peer and Character Nonmental conditions). Research on children in this age range has demonstrated that the mentalizing regions exhibit increasing specificity to mental states compared to nonmental social information from childhood into adulthood (Gweon et al., 2012; Richardson et al., 2020), and thus the age range of our sample may have contributed to our lack of significant Model 3 fit. This interpretation is supported by our behavioral analyses which revealed better accuracy for Mental trials and quicker response for the Peer Mental trials with age (Figure 3 and Table 2). The inclusion of a matched, but fully nonsocial condition would aid in teasing apart the effects of social development.

Another factor potentially contributing to the minimal dissimilarity between mental and nonmental conditions is that the items comprising the Mental condition were deliberately varied to cover a range of mental state categories, including wants, likes, knowledge, beliefs, and emotions, while the Nonmental items asked about activities, physical characteristics, abilities, situations, and possessions (Table S1). Therefore, it is possible that this amount of variation led to nonrepresentative response patterns when averaging across items for each condition per subject. Furthermore, hints for the Mental trials included language about mental states under the assumption that such language would elicit some degree of mentalizing, but it is possible that some participants relied on strategies for making guesses that cut across the Mental/Nonmental conditions, such as semantic associations between hint and response options. Following this interpretation, our behavioral effects for question type might reflect confounding processes related to the Mental condition, such as the inherent abstractness of the language used to describe mental states versus physical attributes, which is particularly challenging to disentangle because the representation of semantic associations, abstract concepts, and social concepts are subserved by overlapping brain regions (e.g., Binney et al., 2016; Xu et al., 2018).

The aforementioned interpretations do not align with the direction of our behavioral findings, nor with some of the activations reported by Alkire et al. (2018). That is, if linguistic abstractness associated with Mental trials was driving our behavioral effects, we would expect slower reaction time and reduced accuracy (Borghi & Zarcone, 2016), rather than the target‐by‐question type interactions in RT and greater accuracy for Mental trials (Table 2). Activations in the dmPFC for the Mental > Nonmental and the Peer Mental > Character Mental contrasts (Alkire et al., 2018) also do not align with prior work that generally implicate lateral cortical structures in the processing of abstract concepts (Binney et al., 2016; Conca et al., 2021; Wang et al., 2010). Moreover, careful examination of our experimental stimuli revealed that a substantial portion of the items could not easily be answered through semantic association, and that for some items, semantic associations would lead to the wrong answer (e.g., “[Target] thinks skiing is dangerous” is semantically related to “Mountain slope” even though the target picking “Hot Cocoa” better matches the target's mental state; Table S1). Nonetheless, the explanations provided above do not preclude the possibility that other, unrealized confounds may have contributed to our lack of Model 3 fit, and future research should work to disentangle the effects of linguistic processes that overlap with social cognition.

Despite these limitations, our findings demonstrate the importance of incorporating realistic, social‐interactive contexts for a better understanding of the neural substrates subserving everyday social cognition, and they highlight the utility of pattern‐similarity‐based analyses for examining the relationship between related social‐cognitive processes. Through this set of approaches, we provided additional evidence for the spontaneous engagement of mentalizing during social interactions, and spotlight the functional contributions of the ATL toward integrating offline social cognition with online social interactions. Future work could build on these findings by establishing more precise neural and behavioral measures of mentalizing and related social‐cognitive processes to better establish process‐level similarities and differences between them. By elucidating the links and dissociations between the various cognitive mechanisms underlying social interactions, we will gain not only a better understanding of the social behaviors that shape our world but also a more complete cognitive map of how different neurocognitive functions relate to each other.

## CONFLICT OF INTEREST

The authors declare that they have no competing interests, financial or otherwise.

## Supporting information


**Appendix S1** Supplementary Information

## Data Availability

The data from consenting participants that support the findings of this study have been uploaded to the National Institute of Mental Health Data Archive (NDA) under collection #2394 and are available upon request at https://nda.nih.gov. Data analysis code can be found here: https://github.com/JunaidMerchant/CMNT_RSA. Group‐level searchlight maps can be found here: https://neurovault.org/collections/ZUBUIWHX/.

## Footnotes

^1^
Throughout the manuscript, we contrast spontaneous with explicit mentalizing, which we define as being explicitly prompted by the task. This is not to be confused with implicit versus explicit cognitive processes (e.g., Frith & Frith, 2008), either of which can be engaged spontaneously.
