# Supplementary material for: Neural similarity between mentalizing and live social interaction during the transition to adolescence
Source: Hum Brain Mapp. 2022 May 12;43(13):4074–90. doi: 10.1002/hbm.25903 (PMC9374881; doi:10.1002/hbm.25903)
Supplement: Supplementary file 1 — Appendix S1 Supplementary Information [file HBM-43-4074-s001.zip › HBM_25903_HBM_25903_SupplementalInformation_FINAL_20220427.pdf]

Table of Contents

Section 1: Additional Methods Reporting **2**

Supplemental Methods 1: Experimental protocol 2

Supplemental Table 1: Experimental stimuli 3

Supplemental Methods 2: Full fMRIprep Report 6

Section 2: Mentalizing/Social Interaction Conjunction ROIs from Alkire and colleagues (2018): RSA Re-Analysis **9**

Supplemental Methods 3: Methods for Conjunction ROI RSA 9

Supplemental Figure 1: Bayesian multilevel (BML) modeling of RSA model fit in conjunction ROIs 10

Supplemental Table 2: Bayesian multilevel (BML) modeling and general linear modeling (GLM) results of RSA model fit in conjunction ROIs 11

Section 3: Assumption Checks and Supplemental Behavioral Analyses **12**

Supplemental Table 3: Descriptive statistics for age and behavioral data 12

Supplemental Figure 2: Distribution plots for age and accuracy measures 12

Supplemental Table 4: Reaction time ANCOVA with age as covariate and gender as a between subject factor 13

Supplemental Table 5: Accuracy ANCOVA with age as covariate and gender as a between subject factor 14

Supplemental Table 6: Non-parametric tests to validate accuracy ANCOVA findings 15

Supplemental Table 7: Post-scan questions 16

Section 4: Supplemental Analyses of Social Interaction ROIs **17**

Supplemental Table 8: Bayesian multilevel (BML) modeling and general linear modeling (GLM) results of RSA model fit in social interaction ROIs from Neurosynth for full sample (n=72) 17

Supplemental Figure 3: Comparison of model fit by number of usable runs within social interaction ROIs 19

Supplemental Table 9: Follow up regressions to examine which condition pair distances contribute to negative relationship between age and Model 2 fit 21

Section 5: Whole-Brain Searchlight Analysis **24**

Supplemental Methods 4: Searchlight analysis 24

Supplemental Figure 4: Searchlight results for Models 1 & 2 25

Supplemental Table 10: Searchlight results for Models 1 & 2 26

Section 6: Reanalysis After Excluding Incorrectly Answered Trials ***26***

Supplemental Methods 5: Reanalysis excluding incorrect trials 26

Supplemental Figure 5: Social interaction ROIs 27

Supplemental Figure 6: Searchlight analysis 28

Supplemental Table 11: Searchlight clusters 29

Supplemental References ***30***

Section 1: Additional Methods Reporting

Supplemental Methods 1: Experimental protocol

The full details of the experimental procedure can be found in the *Task procedures*, sub-section 2.2 (pages 3-4), in the *Materials and Methods* of Alkire and colleagues (2018), and is summarized below for the reader's convenience. Additional details can be found in the Supporting Information page for the Alkire et al., 2018 publication (https://onlinelibrary.wiley.com/doi/suppl/10.1002/hbm.24221):

To create the live illusion, participants were told they would be chatting with a peer in a different laboratory who would also be undergoing an MRI scan. They were told that they would chat with their partners only half the time, and for the other half, they would answer questions provided by a computer about a fictional character of the same gender and age as the participant. After having their photo taken to enhance the live illusion, they were presented with photos of two children that matched the participant's age and gender, and were asked to choose one of the peers to be their chat partner. Images were taken from the NIMH Child Emotional Faces Pictures Set (Egger et al., 2011), Getty Images (www.gettyimages.com), and Google Images search to obtain racial/ethnic diversity that matched participant population.

In the scanner, participants played the role of the "guesser" in a prediction game, and on each trial, they received a one-sentence hint about either their chat partner or a fictional character in a story. Participants answered either "Which will I/she/ he pick?" (Mental) or "Which of these match?" (Non-mental), and chose between two choices via button-press based on the hint provided. Each trial started with a half second cue indicating who they were answering the question about that was the name of the Peer or the Character, followed by a 3.5 second presentation of the hint. This was followed by a 4 second choice period during which the two choice options were presented, which went to the fixation cross once the participant made their choice. After a 2-6 jitter period, the trial ended with a 2 second feedback period during which participants learned whether their choices matched those of the chat partner or the computer (Figure 1 of the main text).

There was a total of 96 trials – half (48 trials) the hints described mental states such as knowledge, beliefs, desires, preferences, and emotions (Mental), and the other half were hints that described facts or situations about the peer or character but made no reference to mental states (Non-mental). Additionally, 48 trials (24 Mental, 24 Non-mental) were presented in the first-person from the perspective of the interaction partner (Peer) and the other 48 in the third-person perspective (Character), creating four conditions – Peer Mental (PM), Peer Non-mental (PNM), Character Mental (CM), and Character Non-mental (CNM) – yielding a fully within-subject 2 (Peer/Character) x 2 (Mental/Non-mental). Individual trials were counterbalanced across participants between Peer and Character conditions. The task was presented using PsychoPy (Peirce, 2009) across four runs of 24 trials each (24 trials per condition total). Trial distribution and inter-trial intervals were optimized using in-lab code that minimized collinearity between events in the design matrix. To maintain the live illusion, the chat partner's photo appeared at the end of every run.

After the scan, participants completed a questionnaire in which they rated on a scale of 1–5 their preference for and attention to the live partner versus the computer. The posttest also probed participants' belief in the live illusion, and any participants expressing disbelief in the live illusion were excluded from analyses.

Supplemental Table 1 includes example stimuli presented to one participant, which includes all the Mental and Non-mental items presented to our participants. The Mental/Non-mental items were counterbalanced across participants such that each item was presented in the Peer and Character conditions approximately an equal number of times throughout data collection. Thus, the examples in the table provide only one of the ways that the Mental/Non-mental items were presented from the perspective of the Peer or Character. Additionally, the spreadsheet contains descriptive statistics about accuracy and reaction time of each item based on our sample of 72 participants.

Supplemental Table 1: Experimental stimuli

Column header descriptions: **Item** - the hint provided to the participant (Note: that each hint was counterbalanced across participants to come from the perspective of the Peer and Character and equal number of times. The form of the hints in this table is from one participant, and does not reflect what every participant saw). Question Type = Whether the item was a Mental or Non-mental (physical item). Correct Answer = What we had tagged as the correct answer. Incorrect Answer = Alternative response choice. Mean Accuracy = Mean accuracy for the item across our sample of 72 participants. Mean RT = Mean response time in seconds for the item across our sample of 72 participants. Med RT = Median response time in seconds for the item across our sample of 72 participants. Stdv RT = Standard deviation of response times for the item across our sample of 72 participants. Omissions = Number of times the item was not answered by a participant.

**Item**	**Question Type**	**Correct Answer**	**Incorrect Answer**	**Mean Accuracy**	**Mean RT**	**Med RT**	**Stdv RT**	**Omiss-ions**

Mia knows her sister is lying.	Mental	Tell on her	Believe her	0.956	2.259	2.326	0.600	1
I am excited to see my friend.	Mental	Hug friend	Ignore friend	1.000	2.167	2.108	0.606	0
Mia wants to borrow a book.	Mental	Library	Mall	0.986	1.631	1.623	0.608	1
Mia likes homemade meals.	Mental	Pack lunch	Buy lunch	0.891	2.135	2.098	0.615	0
Mia does not think the teacher heard her.	Mental	Repeat question	Wait for answer	0.913	2.348	2.372	0.763	1
Mia feels confused during math class.	Mental	Math tutor	Math club	0.879	2.189	2.180	0.682	2
Mia thinks her friend is mad at her.	Mental	Apologize	Annoy friend	0.928	2.190	2.127	0.559	1
Mia wants to stay dry at the lake.	Mental	Picnic	Swimming	0.909	1.935	1.925	0.622	2
I want a healthy snack.	Mental	Carrots	Cookies	0.957	1.578	1.480	0.408	1
I really like to laugh.	Mental	Funny movie	Scary movie	0.986	1.802	1.710	0.544	1
Mia does not know what time it is.	Mental	Clock	Calculator	0.971	1.709	1.668	0.464	0
I want to do some exercise.	Mental	Soccer	Movie	0.884	1.890	1.704	0.565	3
I think flying is fun.	Mental	Plane	Train	0.971	1.625	1.527	0.461	0
I am angry at my mom.	Mental	Glare at mom	Smile at mom	0.879	2.265	2.181	0.663	3
Mia is afraid of heights.	Mental	Rake leaves	Climb tree	0.853	2.109	1.941	0.629	4
Mia is interested in the ocean.	Mental	Aquarium	Zoo	0.970	1.703	1.567	0.477	0
Mia thinks there is candy in the jar.	Mental	Jar	Drawer	0.929	2.167	2.088	0.580	3
I think my friend is busy.	Mental	Leave alone	Ask to hang out	0.712	2.489	2.361	0.692	2
Mia does not like fruit.	Mental	Soda	Juice	0.853	2.324	2.225	0.736	2
I think TV shows should be silly.	Mental	Comedy	Drama	0.910	1.956	1.802	0.719	1
Mia knows her brother is hiding in the closet.	Mental	Open closet	Keep looking	0.851	2.081	2.044	0.529	2
I want to get a good grade.	Mental	Study for test	Watch TV	0.957	1.723	1.563	0.595	1
Mia wants to have fun with her friends.	Mental	Party	Nature walk	0.986	1.942	1.808	0.662	0

Mia wants to listen to loud music.	Mental	Rock	Classical	0.971	1.697	1.660	0.503	0
I like to play with animals.	Mental	Dog	Goldfish	0.956	1.969	1.834	0.540	0
I think skiing is dangerous.	Mental	Hot cocoa	Mountain slope	0.701	2.237	2.123	0.603	4
I know that school is closed today.	Mental	Play outside	Wait at bus stop	0.957	2.130	2.095	0.487	2
I want to eat healthy.	Mental	Fish	Pizza	0.929	1.891	1.834	0.474	1
Mia likes to get exercise.	Mental	Kickball	Card game	0.929	1.828	1.738	0.542	1
I do not think I did well on the test.	Mental	Ask for help	Celebrate	0.985	2.111	2.015	0.624	1
I do not know where I'm going.	Mental	Ask for directions	Walk faster	0.955	2.211	2.146	0.508	1
Mia knows the answer to the question.	Mental	Say answer	Look confused	0.897	2.219	2.154	0.669	5
Mia knows it will rain later.	Mental	Umbrella	Sandals	0.942	1.638	1.525	0.565	3
Mia thinks she is funny.	Mental	Tell a joke	Sing a song	0.970	1.742	1.728	0.582	1
Mia likes to run.	Mental	Soccer	Dodgeball	0.899	2.053	2.083	0.568	3
I am scared of roller coasters.	Mental	Water park	Theme park	0.841	2.053	1.995	0.535	0
Mia likes to read.	Mental	Long novel	Short story	0.941	2.260	2.250	0.577	1
Mia wonders if the weather is nice.	Mental	Window	Basement	0.868	2.239	2.197	0.620	4
I do not want to go to bed early.	Mental	11pm bedtime	7pm bedtime	0.843	2.372	2.173	0.702	3
I know my bike was stolen.	Mental	Call police	Check garage	0.870	2.190	2.157	0.565	1
I care about the environment.	Mental	Recycling	Trash	0.942	1.861	1.666	0.598	1
I think it will be warm today.	Mental	T-shirt	Long sleeves	0.838	1.892	1.836	0.596	1
I know the house is empty.	Mental	Leave a note	Knock on door	0.831	2.462	2.500	0.658	3
Mia wants to watch a true story.	Mental	History Channel	Cartoon Network	0.928	2.064	1.985	0.580	1
Mia worries about being in front of people.	Mental	Read a book	Give a speech	0.758	2.129	2.062	0.582	3
I want to save money.	Mental	Yo-yo	Video game	0.621	2.505	2.389	0.624	5

I like calm, relaxing rides.	Mental	Ferris wheel	Roller coaster	0.941	2.155	2.126	0.538	3
I am bored.	Mental	New game	Chores	0.928	2.289	2.164	0.680	1
My bedroom gets cold at night.	Non-mental	Close window	Open window	0.818	2.260	2.111	0.595	2
My headphones are broken.	Non-mental	Draw a picture	Listen to music	0.803	2.247	2.149	0.634	4
I am in the living room.	Non-mental	Watch TV	Cook dinner	0.900	2.107	1.847	0.708	3
I am going to a birthday party.	Non-mental	Party hat	Reading glasses	0.956	2.072	1.960	0.643	2
Mia's yard does not have any trees.	Non-mental	Volleyball net	Treehouse	0.851	2.179	2.045	0.591	1
I can kick the ball far.	Non-mental	Soccer	Basketball	0.986	1.732	1.621	0.626	1
Mia is going to a water park.	Non-mental	Bathing suit	Athletic shorts	0.886	2.093	2.014	0.630	2
I can't see things far away.	Non-mental	Glasses	Blindfold	0.958	1.912	1.777	0.716	1
Mia's paint palette is dirty.	Non-mental	Clean palette	Paint picture	0.690	2.184	1.977	0.634	6
Mia is in a research lab.	Non-mental	Pay attention	Jump up and down	0.922	2.331	2.215	0.666	2
My dog is sick.	Non-mental	Veterinarian	Doctor	0.913	1.762	1.704	0.593	2
I am drawing something blue.	Non-mental	The ocean	The mountains	0.956	1.721	1.626	0.457	1
Mia is going skiing.	Non-mental	Snow boots	Tennis shoes	0.912	2.105	2.030	0.596	3
Mia is in the kitchen.	Non-mental	Get a snack	Go to sleep	0.941	1.859	1.778	0.546	3
Mia shares a room with three siblings.	Non-mental	Bunk beds	Single beds	0.868	2.227	1.985	0.782	5
I came in third place in the contest.	Non-mental	Bronze medal	Gold medal	0.859	2.149	2.200	0.642	1
Mia's computer screen is very dark.	Non-mental	Brightness	Volume	0.868	2.228	2.193	0.687	2
Mia stayed home sick from school today.	Non-mental	Doctor's office	Math test	0.939	2.221	2.047	0.694	1
I live very close to the pool.	Non-mental	Swim team	Soccer team	0.912	1.842	1.836	0.571	3
Mia is playing with three friends.	Non-mental	Card game	Chess	0.870	2.416	2.296	0.693	0

I can speak Spanish.	Non-mental	Mexico	Japan	0.868	1.967	1.808	0.670	1
I sprained my ankle.	Non-mental	Watch a movie	Go for a hike	0.814	2.250	2.143	0.639	3
I am not on vacation.	Non-mental	Dresser	Suitcase	0.818	2.444	2.460	0.691	2
I have to carry a lot of books.	Non-mental	Backpack	Plastic bag	0.929	1.948	1.893	0.586	2
I just got a new sleeping bag.	Non-mental	Go camping	Go swimming	0.957	1.837	1.730	0.605	1
Mia is running late for her piano lesson.	Non-mental	Shortcut	Long way	0.912	2.194	2.260	0.652	1
I have shin guards.	Non-mental	Soccer	Fishing	0.925	1.582	1.469	0.590	0
I do not have any paper.	Non-mental	Sidewalk chalk	Colored pencils	0.754	2.568	2.495	0.643	6
I have ten empty glass bottles.	Non-mental	Recycling	Trash	0.969	2.087	1.985	0.592	2
Mia has a lot of clothes.	Non-mental	Big closet	Small closet	0.956	1.994	1.893	0.603	1
I am going on a hike.	Non-mental	Trail mix	Soup	0.941	2.206	2.100	0.676	2
Mia has a kite.	Non-mental	Windy day	Rainy day	0.926	2.072	1.954	0.631	2
I have a sore throat.	Non-mental	Cough drop	Tissues	0.941	2.099	1.985	0.584	0
Mia can play the piano and violin.	Non-mental	Music	Art	0.938	1.601	1.550	0.460	1
Mia does not own a helmet.	Non-mental	Jogging	Bike ride	0.824	2.057	1.827	0.678	2
I invited a lot of friends to the party.	Non-mental	Laser tag	Sleepover	0.629	2.575	2.572	0.572	4
Mia is not at the pool.	Non-mental	Swing set	Diving board	0.809	2.273	2.255	0.622	8
Mia is wearing a costume.	Non-mental	School play	Spelling bee	0.868	2.303	2.247	0.520	3
I only ride my bike in the street.	Non-mental	Road bike	Mountain bike	0.928	2.234	2.074	0.559	1
Mia can do the splits.	Non-mental	Gymnastics	Singing	0.986	1.589	1.471	0.567	0
Mia is inside of a tent.	Non-mental	Camping	Sports	0.956	1.700	1.540	0.665	0
Mia is playing with her little brother.	Non-mental	Nerf gun	Homework	0.942	2.134	2.095	0.560	2

I have big feet.	Non-mental	Big shoes	Small shoes	0.985	1.652	1.562	0.481	0
Mia plays Minecraft.	Non-mental	Computer	Mining pick	0.667	2.246	2.286	0.741	9
I am going to the beach later today.	Non-mental	Sunscreen	Snowboard	0.986	1.820	1.752	0.400	1
Mia is very tall for her age.	Non-mental	Basketball	Gymnastics	0.841	1.936	1.981	0.480	4
Mia lives far away from school.	Non-mental	Bus	Bike	0.956	1.900	1.834	0.565	1
Mia is wearing socks today.	Non-mental	Shoes	Sandals	0.887	2.078	2.116	0.549	3

Supplemental Methods 2: Full fMRIprep Report

Results included in this manuscript come from preprocessing performed using *fMRIPrep* 1.4.1 (Esteban, Markiewicz, et al. (2018); Esteban, Blair, et al. (2018); RRID:SCR_016216), which is based on *Nipype* 1.2.0 (Gorgolewski et al. (2011); Gorgolewski et al. (2018); RRID:SCR_002502).

Anatomical data preprocessing. The T1-weighted (T1w) image was corrected for intensity non-uniformity (INU) with N4BiasFieldCorrection (Tustison et al. 2010), distributed with ANTs 2.2.0 (Avants et al. 2008, RRID:SCR_004757), and used as T1w-reference throughout the workflow. The T1w-reference was then skull-stripped with a *Nipype* implementation of the antsBrainExtraction.sh workflow (from ANTs), using MNI152NLin2009cAsym as target template. Brain tissue segmentation of cerebrospinal fluid (CSF), white-matter (WM) and gray-matter (GM) was performed on the brain-extracted T1w using fast (FSL 5.0.9, RRID:SCR_002823, Zhang, Brady, and Smith 2001). Brain surfaces were reconstructed using recon-all (FreeSurfer 6.0.1, RRID:SCR_001847, Dale, Fischl, and Sereno 1999), and the brain mask estimated previously was refined with a custom variation of the method to reconcile ANTs-derived and FreeSurfer-derived segmentations of the cortical gray-matter of Mindboggle (RRID:SCR_002438, Klein et al. 2017). Volume-based spatial normalization to three standard spaces (MNIPediatricAsym, MNI152NLin2009cAsym, MNI152NLin6Asym) was performed through nonlinear registration with antsRegistration (ANTs 2.2.0), using brain-extracted versions of both T1w reference and the T1w template. The following templates were selected for spatial normalization: *MNI's unbiased standard MRI template for pediatric data from the 4.5 to 18.5y age range* [(**???**), RRID:SCR_008796; TemplateFlow ID: MNIPediatricAsym], *ICBM 152 Nonlinear Asymmetrical template version 2009c* [Fonov et al. (2009), RRID:SCR_008796; TemplateFlow ID: MNI152NLin2009cAsym], *FSL's MNI ICBM 152 non-linear 6th Generation Asymmetric Average Brain Stereotaxic Registration Model* [Evans et al. (2012), RRID:SCR_002823; TemplateFlow ID: MNI152NLin6Asym].

Functional data preprocessing. For each of the 4 BOLD runs found per subject (across all tasks and sessions), the following preprocessing was performed. First, a reference volume and its skull-stripped version were generated using a custom methodology of *fMRIPrep*. A deformation field to correct for susceptibility distortions was estimated based on *fMRIPrep*'s *fieldmap-less* approach. The deformation field is that resulting from co-registering the BOLD reference to the same-subject T1w-reference with its intensity inverted (Wang et al. 2017; Huntenburg 2014). Registration is performed with antsRegistration (ANTs 2.2.0), and the process regularized by constraining deformation to be nonzero only along the phase-encoding direction, and modulated with an average fieldmap template (Treiber et al. 2016). Based on the estimated susceptibility distortion, an unwarped BOLD reference was calculated for a more accurate co-registration with the anatomical reference. The BOLD reference was then co-registered to the T1w reference using bbregister (FreeSurfer) which implements boundary-based registration (Greve and Fischl 2009). Co-registration was configured with nine degrees of freedom to account for distortions remaining in the BOLD reference. Head-motion parameters with respect to the BOLD reference (transformation matrices, and six corresponding rotation and translation parameters) are estimated before any spatiotemporal filtering using mcflirt (FSL 5.0.9, Jenkinson et al. 2002). BOLD runs were slice-time corrected using 3dTshift from AFNI 20160207 (Cox and Hyde 1997, RRID:SCR_005927). The BOLD time-series were resampled to surfaces on the following spaces: *fsaverage5*. The BOLD time-series (including slice-timing correction when applied) were resampled onto their original, native space by applying a single, composite transform to correct for head-motion and susceptibility distortions. These resampled BOLD time-series will be referred to as *preprocessed BOLD in original space*, or just *preprocessed BOLD*. The BOLD time-series were resampled into several standard spaces, correspondingly generating the following *spatially-normalized, preprocessed BOLD runs*: MNIPediatricAsym, MNI152NLin2009cAsym, MNI152NLin6Asym. First, a reference volume and its skull-stripped version were generated using a custom methodology of *fMRIPrep*. Automatic removal of motion artifacts using independent component analysis (ICA-AROMA, Pruim et al. 2015) was performed on the *preprocessed BOLD on MNI space* time-series after removal of non-steady state volumes and spatial smoothing with an isotropic, Gaussian kernel of 6mm FWHM (full-width half-maximum). Corresponding "non-aggresively" denoised runs were produced after such smoothing. Additionally, the "aggressive" noise-regressors were collected and placed in the corresponding confounds file. Several confounding time-series were calculated based on the *preprocessed BOLD*: framewise displacement (FD), DVARS and three region-wise global signals. FD and DVARS are calculated for each functional run, both using their implementations in *Nipype* (following the definitions by Power et al. 2014). The three global signals are extracted within the CSF, the WM, and the whole-brain masks. Additionally, a set of physiological regressors were extracted to allow for component-based noise correction (*CompCor*, Behzadi et al. 2007). Principal components are estimated after high-pass filtering the *preprocessed BOLD* time-series (using a discrete cosine filter with 128s cut-off) for the two *CompCor* variants: temporal (tCompCor) and anatomical (aCompCor). tCompCor components are then calculated from the top 5% variable voxels within a mask covering the subcortical regions. This subcortical mask is obtained by heavily eroding the brain mask, which ensures it does not include cortical GM regions. For aCompCor, components are calculated within the intersection of the aforementioned mask and the union of CSF and WM masks calculated in

T1w space, after their projection to the native space of each functional run (using the inverse BOLD-to-T1w transformation). Components are also calculated separately within the WM and CSF masks. For each CompCor decomposition, the *k* components with the largest singular values are retained, such that the retained components' time series are sufficient to explain 50 percent of variance across the nuisance mask (CSF, WM, combined, or temporal). The remaining components are dropped from consideration. The head-motion estimates calculated in the correction step were also placed within the corresponding confounds file. The confound time series derived from head motion estimates and global signals were expanded with the inclusion of temporal derivatives and quadratic terms for each (Satterthwaite et al. 2013). Frames that exceeded a threshold of 0.5 mm FD or 1.5 standardised DVARS were annotated as motion outliers. All resamplings can be performed with *a single interpolation step* by composing all the pertinent transformations (i.e. head-motion transform matrices, susceptibility distortion correction when available, and co-registrations to anatomical and output spaces). Gridded (volumetric) resamplings were performed using antsApplyTransforms (ANTs), configured with Lanczos interpolation to minimize the smoothing effects of other kernels (Lanczos 1964). Non-gridded (surface) resamplings were performed using mri_vol2surf (FreeSurfer).

Many internal operations of *fMRIPrep* use *Nilearn* 0.5.2 (Abraham et al. 2014, RRID:SCR_001362), mostly within the functional processing workflow. For more details of the pipeline, see the section corresponding to workflows in *fMRIPrep*'s documentation.

Section 2: Mentalizing/Social Interaction Conjunction ROIs from Alkire and colleagues (2018): RSA Re-Analysis

Supplemental Methods 3: Methods for Conjunction ROI RSA

To evaluate if overlapping brain activations between social interaction (without explicit mentalizing demands) and offline mentalizing reported by Alkire and colleagues (2018) are indicative of similar underlying representations, we conducted RSA in each of the four conjunction ROIs they present in Figure 5/Table 2 (page 10), which includes: 1) left anterior temporal lobe (ATL) cluster extending into the insula and lateral OFC; 2) right ATL; 3) right posterior superior temporal sulcus (pSTS); 4) right inferior frontal gyrus (IFG). This preliminary analysis served both as a proof-of-concept for the sensitivity of RSA to evaluate the relationship between mentalizing and social interaction, which motivated the primary analyses reported in the main text, and provided a means to assess the interpretations made by Alkire and

colleagues (2018) about overlapping brain activations in a larger sample of participants who underwent the same experimental procedure.

To this end, we followed the same procedure for the model-based RSA utilized in the main text. Neural representational dissimilarity matrices (RDMs) were calculated per subject for each ROI by extracting voxel-wise *t*-values associated with each condition, and calculating the Euclidean distance between each pair of conditions. Neural RDMs were tested against three conceptual models of hypothesized relationships between conditions, which were formalized as binary RDMs wherein 0=similar and 1=dissimilar. Model 1, or the "*interaction engages mentalizing*" model, assumes that Character Mental and both Peer conditions are similar (i.e., have 0's) with each other and dissimilar (i.e., have 1's) from Character Non-mental. Model 2, or the "*interaction*" model, assumes that both Peer conditions are similar to each other and both Character conditions are similar to each other, but Peer and Character conditions are dissimilar from each other. Model 3, or the "*mentalizing*" model, assumes that both Mental conditions are similar to each other and both Non-mental conditions are similar to each other, but Mental and Non-mental conditions are dissimilar from each other. Model fit was estimated by calculating Kendall's Tau-a rank-order correlation coefficient between the off-diagonal elements of the neural and model RDMs. For each ROI, model fit was calculated between each participant's neural RDM and the three model RDMs, and were z-transformed before being entered into bayesian multilevel (BML) model using AFNI's RBA program (Chen et al., 2019). This approach provides quantitative estimates for the strength of evidence in favor of each model per ROI, and confers several advantages over traditional null hypothesis significance testing (Chen et al., 2021).

Supplemental Figure 1: Bayesian multilevel (BML) modeling of RSA model fit in conjunction ROIs

A) The four conjunction ROIs are depicted: left anterior temporal lobe (LATL; red), right anterior temporal lobe (RATL; green), right posterior superior temporal sulcus (RpSTS; yellow), and right inferior frontal gyrus (RIFG; blue). **B)** BML model results of model fit to our three conceptual models to each of our four ROIs ordered by posterior probability (P^+). Colors represent values of P^+: the posterior probability that the effect is greater than 0. The analysis revealed that four ROIs exhibited strong evidence in favor of a positive effect: Model 1 fit in right pSTS and left ATL, Model 2 fit in the left ATL and right IFG. Regions showing strong evidence of model fit that fall within the positive 95% quantile interval under BML, which corresponds with a two-tailed p-value of 0.05 under conventional statistical testing framework. **C)** For comparison of the model fit estimates obtained from the original sample of 28 reported in Alkire et al. (2018; in green), the sample of 44 new participants included in the current study (in yellow), and the full sample of 73 (in gray). Results show comparable effects across subgroups.

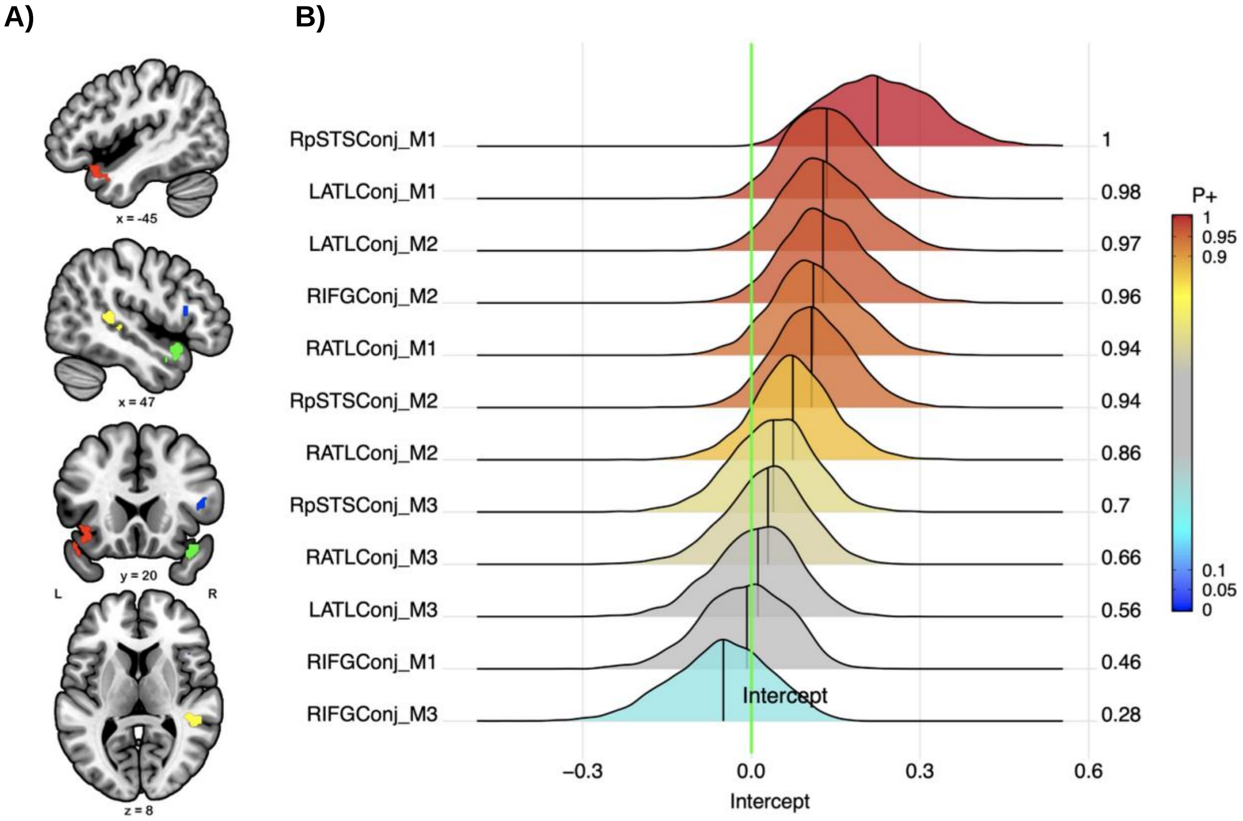

A)
B)
x = -45
x = 47
y = 20
L
R
z = 8
RpSTSConj_M1
LATLConj_M1
LATLConj_M2
RIFGConj_M2
RATLConj_M1
RpSTSConj_M2
RATLConj_M2
RpSTSConj_M3
RATLConj_M3
LATLConj_M3
RIFGConj_M1
RIFGConj_M3
1
0.98
0.97
0.96
0.94
0.94
0.86
0.7
0.66
0.56
0.46
0.28
Intercept
−0.3
0.0
0.3
0.6
Intercept
P+
1
0.95
0.9
0.1
0.05
0

C)

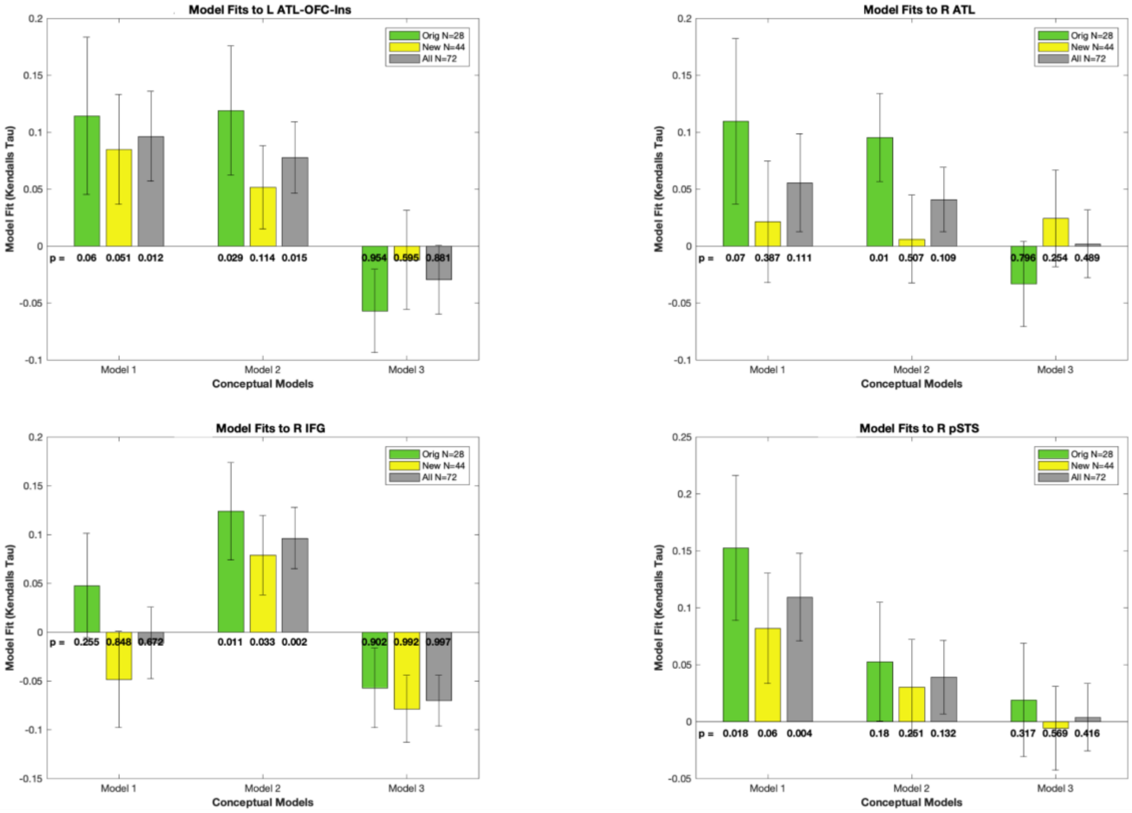

Supplemental Table 2: Bayesian multilevel (BML) modeling and general linear modeling (GLM) results of RSA model fit in conjunction ROIs

These tables report the full statistics from the BML

A) Summary of region effects for the intercept under BML.

ROI x Model	mean	SD	P+	2.50%	5%	50%	95%	97.50%
LATL - Model 1	0.1378	0.0753	0.9760	0.0015	0.0212	0.1340	0.2695	0.2969
LATL - Model 2	0.1321	0.0771	0.9663	-0.0099	0.0119	0.1275	0.2652	0.2910
LATL - Model 3	0.0075	0.0771	0.5589	-0.1538	-0.1235	0.0116	0.1278	0.1493
RATL - Model 1	0.1134	0.0762	0.9394	-0.0320	-0.0057	0.1100	0.2418	0.2667
RATL - Model 2	0.0746	0.0727	0.8623	-0.0719	-0.0436	0.0737	0.1960	0.2212

RATL - Model 3	0.0255	0.0753	0.6566	-0.1344	-0.1043	0.0294	0.1428	0.1634
RIFG - Model 1	-0.0120	0.0790	0.4580	-0.1754	-0.1477	-0.0080	0.1084	0.1275
RIFG - Model 2	0.1320	0.0774	0.9637	-0.0119	0.0112	0.1272	0.2665	0.2914
RIFG - Model 3	-0.0548	0.0892	0.2791	-0.2396	-0.2053	-0.0498	0.0853	0.1039
RpSTS - Model 1	0.2272	0.0938	0.9994	0.0611	0.0802	0.2241	0.3859	0.4156
RpSTS - Model 2	0.1088	0.0710	0.9437	-0.0254	-0.0023	0.1071	0.2285	0.2525
RpSTS - Model 3	0.0361	0.0753	0.6963	-0.1193	-0.0917	0.0390	0.1516	0.1775

B) Summary of region effects for the intercept under GLM (for comparison).

ROI x Model	mean	SD	2-sided p-value	2.50%	5%	50%	95%	97.50%
LATL - Model 1	0.1879	0.1154	0.1079	-1.2781	-0.0044	0.1879	0.9163	0.4179
LATL - Model 2	0.1774	0.0907	0.0544	-0.9751	0.0263	0.1774	0.7501	0.3583
LATL - Model 3	-0.0522	0.0825	0.5292	-1.1008	-0.1897	-0.0522	0.4689	0.1124
RATL - Model 1	0.1409	0.1163	0.2298	-1.3370	-0.0529	0.1409	0.8753	0.3728
RATL - Model 2	0.0731	0.0903	0.4212	-1.0743	-0.0774	0.0731	0.6432	0.2531
RATL - Model 3	-0.0157	0.0842	0.8531	-1.0858	-0.1560	-0.0157	0.5161	0.1523
RIFG - Model 1	-0.0835	0.1020	0.4156	-1.3790	-0.2534	-0.0835	0.5603	0.1198
RIFG - Model 2	0.1774	0.0876	0.0466	-0.9358	0.0314	0.1774	0.7306	0.3521
RIFG - Model 3	-0.1566	0.0668	0.0219	-1.0051	-0.2679	-0.1566	0.2651	-0.0234
RpSTS - Model 1	0.3392	0.1068	0.0022	-1.0183	0.1611	0.3392	1.0138	0.5522
RpSTS - Model 2	0.1357	0.0967	0.1649	-1.0930	-0.0255	0.1357	0.7462	0.3285
RpSTS - Model 3	0.0000	0.0790	1.0000	-1.0038	-0.1317	0.0000	0.4988	0.1575

Section 3: Assumption Checks and Supplemental Behavioral Analyses

Supplemental Table 3: Descriptive statistics for age and behavioral data

Descriptive statistics for age of our sample, and accuracy and reaction time measures for the Character Mental (CM), Character Non-mental (CNM), Peer Mental (PM), and Peer Non-mental (PNM) conditions. Statistics were used to evaluate the assumption of normality for the repeated measures analyses of co-variance (ANCOVA) reported in the main text. Because the Shapiro-Wilk tests for normality indicate violations of the assumption of normality in the accuracy measures, distribution plots for these measures before and after transformation are presented in Supplemental Figure 2.

	**Mean Accuracy (proportion of correct answers)**				**Mean Reaction Time (seconds)**			
**Descriptive Statistics**	**CM**	**CNM**	**PM**	**PNM**	**CM**	**CNM**	**PM**	**PNM**
**Median**	0.917	0.875	0.917	0.917	2.123	2.087	2.016	2.057
**Mean**	0.9	0.881	0.91	0.896	2.096	2.11	2.004	2.036
**Std. Error of Mean**	0.01	0.01	0.009	0.01	0.035	0.036	0.036	0.036
**Variance**	0.007	0.007	0.006	0.007	0.088	0.095	0.095	0.095
**Skewness**	-0.575	-0.525	-0.943	-0.878	-0.279	-0.353	-0.113	-0.331
**Std. Error of Skewness**	0.283	0.283	0.283	0.283	0.283	0.283	0.283	0.283
**Kurtosis**	-0.722	-0.218	0.944	0.168	0.347	0.425	0.591	0.762
**Std. Error of Kurtosis**	0.559	0.559	0.559	0.559	0.559	0.559	0.559	0.559
**Shapiro-Wilk**	0.906	0.945	0.902	0.901	0.985	0.984	0.991	0.989
**P-value of Shapiro-Wilk**	< .0001	0.004	< .0001	< .0001	0.54	0.486	0.891	0.803

Supplemental Figure 2: Distribution plots for age and accuracy measures

Top row shows density for age and the accuracy scores for each simple condition – Character Mental (CM), Character Non-mental (CNM), Peer Mental (PM), and Peer Non-mental (PNM) – before data transformations. The bottom row shows density for age after log transformation, and for the accuracy scores after an arcsine transformation (the recommended transformation for proportion and percentage data). Arcsine transformations of the accuracy data did not bring the data to an acceptible level of normality for ANOVA. Therefore, accuracy ANCOVAs were conducted in the main text using untransformed data, the results for which were validated using non-parametric tests that are reported in Supplemental Table 6.

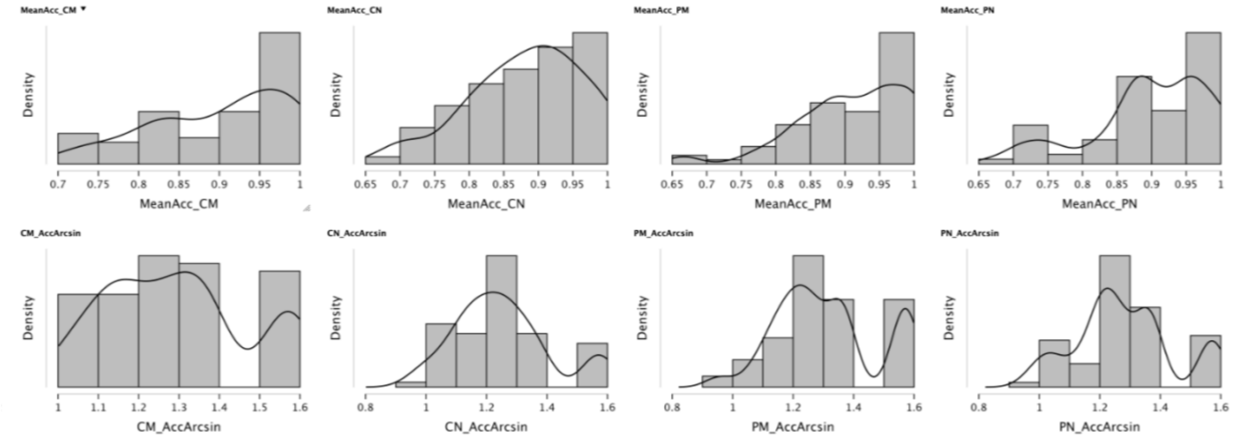

Supplemental Table 4: Reaction time ANCOVA with age as covariate and gender as a between subject factor

2 (target: Peer/Character) x 2 (question type: Mental/Non-mental) repeated measures ANCOVA results for reaction time with log-transformed age as between subject covariate, and gender as between subject factor.

**Within Subjects Effects**	**Sum of Squares**	**df**	**Mean Square**	**F**	**p**	**η²**
Target	0.005	1	0.005	0.465	0.498	2.01E-04
Target ✻ Gender	0.005	1	0.005	0.43	0.514	1.86E-04
Target ✻ Age	0.034	1	0.034	2.923	0.092	0.001
Residuals	0.81	69	0.012			

Question Type	0.016	1	0.016	1.475	0.229	5.84E-04
Question Type ✻ Gender	0.002	1	0.002	0.171	0.681	6.76E-05
Question Type ✻ Age	0.025	1	0.025	2.349	0.13	9.30E-04
Residuals	0.742	69	0.011			
Target ✻ Question Type	0.05	1	0.05	4.086	0.047	0.002
Target ✻ Question Type ✻ gender	1.88E-04	1	1.88E-04	0.015	0.902	6.90E-06
Target ✻ Question Type ✻ Age	0.056	1	0.056	4.595	0.036	0.002
Residuals	0.84	69	0.012			
**Between Subjects Effects**						
Gender	0.702	1	0.702	2.515	0.117	0.026
Age	4.625	1	4.625	16.576	1.23E-04	0.17
Residuals	19.25	69	0.279			
Note. Type III Sum of Squares						

**Descriptives**					
**Target**	**Question Type**	**Gender**	**Mean**	**SD**	**N**
Character	Mental	F	2.057	0.236	28
		M	2.121	0.33	44
	Non-Mental	F	2.078	0.245	28
		M	2.131	0.343	44
Peer	Mental	F	1.988	0.253	28
		M	2.015	0.342	44
	Non-Mental	F	2.015	0.256	28
		M	2.049	0.341	44

Supplemental Table 5: Accuracy ANCOVA with age as covariate and gender as a between subject factor

2 (target: Peer/Character) x 2 (question type: Mental/Non-mental) repeated measures ANCOVA results for accuracy with age as between subject covariate, and gender as between subject factor.

**Within Subjects Effects**	**Sum of Squares**	**df**	**Mean Square**	**F**	**p**	**η²**
Target	0.01	1	0.01	2.879	0.094	5.00E-03
Target ∗ Gender	0.004	1	0.004	1.23	0.271	2.00E-03
Target ∗ Age	0.007	1	0.007	1.907	0.172	0.003
Residuals	0.247	69	0.004			
Question Type	0.012	1	0.012	4.14	0.046	6.00E-03
Question Type ∗ Gender	0.001	1	0.001	0.4	0.529	5.96E-04
Question Type ∗ Age	0.017	1	0.017	5.884	0.018	9.00E-03
Residuals	0.203	69	0.003			
Target ∗ Question Type	0.005	1	0.005	1.808	0.183	0.003
Target ∗ Question Type ∗ gender	3.00E-03	1	3.00E-03	0.887	0.35	1.00E-03
Target ∗ Question Type ∗ Age	0.005	1	0.005	1.769	0.188	0.003
Residuals	0.195	69	0.003			
**Between Subjects Effects**						
Gender	0.023	1	0.023	1.299	0.258	0.011
Age	0.04	1	0.04	2.314	1.33E-01	0.02
Residuals	1.205	69	0.017			

Note. Type III Sum of Squares						

**Descriptives**					
**Target**	**Question Type**	**Gender**	**Mean**	**SD**	**N**
Character	Mental	F	0.894	0.085	28
		M	0.903	0.086	44
	Non-Mental	F	0.89	0.081	28
		M	0.876	0.084	44
Peer	Mental	F	0.922	0.057	28
		M	0.902	0.089	44
	Non-Mental	F	0.913	0.07	28
		M	0.885	0.092	44

Supplemental Table 6: Non-parametric tests to validate accuracy ANCOVA findings

**Friedman Test**				
**Factor**	**Chi-Squared**	**df**	**p**	**Kendall's W**
Target	2.045	1	0.153	0.013
Question Type	2.539	1	0.111	0.02

**Paired Samples T-Test**				
**Measure 1**	**Measure 2**	**W**	**p**	**Rank-Biserial Correlation**
CM	CNM	1082	0.042	1082
PM	PNM	879.5	0.146	879.5

CM	PM	593.5	0.28	593.5
PNM	PNM	615.5	0.194	615.5
Character	Peer	792	0.196	792
Mental	Non-mental	1217	0.026	1217
Note. Wilcoxon signed-rank test.				

**Spearman's Correlations**		
Mental	Spearman's rho	0.281
	p-value	0.017
Non-mental	Spearman's rho	0.082
	p-value	0.494
Mental - Non-mental	Spearman's rho	0.291
	p-value	0.013

Supplemental Table 7: Post-scan questions

Participants were asked to rate on a scale from 1-5 how much they liked chatting with their Peer and when answering questions about the Character (1=not at all, 5=a lot); how much attention they paid when chatting with their Peer and when answering questions about the Character (1=not at all, 5=a lot); how much attention they liked making guesses when chatting with their Peer and when answering questions about the Character (1=not at all, 5=a lot); how hard it was to make guesses when chatting with their Peer and when answering questions about the Character (1=very easy, 5=very hard). Because the response data violate the assumptions of normality, results from parametric and non-parametric tests are reported. Results indicate significantly higher liking, attention, and guessing enjoyment for the Peer versus the Character conditions, but that they found it equally difficult to make guesses in both conditions.

Paired Samples T-Test

**Measure 1**	**Measure 2**	**Test**	**Statistic**	**df**	**p**	**Effect Size**
Peer like	Character like	Student	9.234	71	8.69E-14	1.088

		Wilcoxon	1987.5		1.28E-09	0.853
Peer attention	Character attention	Student	5.152	71	2.22E-06	0.607
		Wilcoxon	1076		1.03E-05	0.688
Peer like to guess	Character like to guess	Student	4.213	71	7.29E-05	0.497
		Wilcoxon	799.5		1.91E-04	0.615
Peer hard guess	Character hard guess	Student	0.448	71	0.655	0.053
		Wilcoxon	361		0.654	0.084

Note. For the Student t-test, effect size is given by Cohen's *d* . For the Wilcoxon test, effect size is given by the matched rank biserial correlation.

**Descriptives**	**N**	**Mean**	**SD**	**SE**
Peer like	72	4.25	0.783	0.092
Char like	72	2.778	0.996	0.117
Peer attention	72	3.931	0.924	0.109
Char attention	72	3.292	0.985	0.116
Peer like guess	72	3.819	0.939	0.111
Char like guess	72	3.319	1.032	0.122
Peer hard guess	72	2.264	1.163	0.137
Char hard guess	72	2.194	1.285	0.151

Section 4: Supplemental Analyses of Social Interaction ROIs

Supplemental Table 8: Bayesian multilevel (BML) modeling and general linear modeling (GLM) results of RSA model fit in social interaction ROIs from Neurosynth for full sample (n=72)

A) The mean and standard deviation (SD) of model fit estimates from BML reported alongside the posterior probability (P^{+}), which quantifies the evidence that the effect is greater than 0. The remaining columns are estimates of the value of the posterior distribution value for important quantiles.

A) Summary of region effects for the intercept under BML.

ROI x Model	mean	SD	P+	2.50%	5%	50%	95%	97.50%
dmPFC - Model 1	0.0611	0.0773	0.7825	-0.0939	-0.0686	0.0614	0.1865	0.2086
dmPFC - Model 2	0.2016	0.0787	0.9960	0.0459	0.0716	0.2009	0.3340	0.3598
dmPFC - Model 3	0.0008	0.0763	0.5090	-0.1505	-0.1256	0.0017	0.1246	0.1520
lATL - Model 1	0.1750	0.0840	0.9850	0.0187	0.0412	0.1737	0.3210	0.3441
lATL - Model 2	0.0237	0.0749	0.6275	-0.1273	-0.1036	0.0249	0.1446	0.1621
lATL - Model 3	0.0552	0.0771	0.7605	-0.0991	-0.0745	0.0561	0.1826	0.2020
lCaudate - Model 1	0.0575	0.0761	0.7825	-0.0902	-0.0685	0.0566	0.1846	0.2054
lCaudate - Model 2	0.1296	0.0767	0.9555	-0.0165	0.0031	0.1275	0.2619	0.2837
lCaudate - Model 3	-0.0668	0.0842	0.2135	-0.2317	-0.2089	-0.0640	0.0746	0.1011
lCrblm - Model 1	0.0025	0.0781	0.5245	-0.1523	-0.1271	0.0046	0.1271	0.1522
lCrblm - Model 2	0.1509	0.0743	0.9780	0.0069	0.0278	0.1493	0.2714	0.2953
lCrblm - Model 3	-0.1069	0.0823	0.0945	-0.2787	-0.2518	-0.1046	0.0239	0.0411
lTPJ - Model 1	0.1917	0.0798	0.9940	0.0450	0.0658	0.1885	0.3242	0.3539
lTPJ - Model 2	0.2138	0.0799	0.9990	0.0598	0.0854	0.2120	0.3443	0.3697

lTPJ - Model 3	-0.0127	0.0814	0.4380	-0.1792	-0.1509	-0.0123	0.1202	0.1445
pgACC - Model 1	0.0201	0.0770	0.6125	-0.1364	-0.1080	0.0204	0.1485	0.1709
pgACC - Model 2	0.2200	0.0814	0.9950	0.0650	0.0934	0.2186	0.3549	0.3848
pgACC - Model 3	-0.0555	0.0809	0.2505	-0.2189	-0.1897	-0.0574	0.0825	0.0984
rATL - Model 1	0.2201	0.0862	0.9990	0.0579	0.0809	0.2189	0.3656	0.3861
rATL - Model 2	0.1027	0.0739	0.9155	-0.0387	-0.0199	0.1045	0.2207	0.2466
rATL - Model 3	0.0185	0.0772	0.6000	-0.1402	-0.1085	0.0173	0.1456	0.1663
rCrblm - Model 1	0.1491	0.0800	0.9745	-0.0001	0.0224	0.1445	0.2865	0.3138
rCrblm - Model 2	0.0807	0.0762	0.8590	-0.0728	-0.0477	0.0814	0.2006	0.2235
rCrblm - Model 3	-0.0387	0.0779	0.3290	-0.1913	-0.1683	-0.0365	0.0824	0.1039
rITG - Model 1	0.1440	0.0770	0.9680	-0.0082	0.0206	0.1433	0.2704	0.2946
rITG - Model 2	0.0997	0.0768	0.9125	-0.0558	-0.0293	0.0979	0.2310	0.2582
rITG - Model 3	-0.0921	0.0820	0.1280	-0.2605	-0.2332	-0.0892	0.0368	0.0590
rTPJ - Model 1	0.1873	0.0818	0.9895	0.0282	0.0582	0.1864	0.3224	0.3432
rTPJ - Model 2	0.2669	0.0865	1.0000	0.0980	0.1310	0.2679	0.4081	0.4364
rTPJ - Model 3	-0.1296	0.0829	0.0590	-0.2935	-0.2695	-0.1273	0.0055	0.0272
rvlPFC - Model 1	0.1038	0.0775	0.9110	-0.0505	-0.0246	0.1042	0.2303	0.2552
rvlPFC - Model 2	0.1133	0.0752	0.9335	-0.0362	-0.0116	0.1131	0.2380	0.2640
rvlPFC - Model 3	0.0696	0.0766	0.8175	-0.0808	-0.0546	0.0691	0.1979	0.2236
sgACC - Model 1	0.0051	0.0768	0.5345	-0.1510	-0.1259	0.0057	0.1295	0.1486
sgACC - Model 2	0.0087	0.0811	0.5535	-0.1555	-0.1247	0.0089	0.1391	0.1660
sgACC - Model 3	0.0389	0.0780	0.6910	-0.1081	-0.0878	0.0373	0.1669	0.1931
vmPFC - Model 1	0.0092	0.0767	0.5570	-0.1460	-0.1233	0.0117	0.1320	0.1508
vmPFC - Model 2	0.1126	0.0738	0.9370	-0.0316	-0.0066	0.1133	0.2341	0.2619

vmPFC - Model 3	-0.0325	0.0777	0.3355	-0.1882	-0.1616	-0.0303	0.0918	0.1161

B) Summary of region effects for the intercept under GLM (for comparison).

ROI x Model	mean	SD	2-sidedp	2.50%	5%	50%	95%	97.50%
dmPFC - Model 1	0.0522	0.1057	0.6231	-1.2910	-0.1240	0.0522	0.7196	0.2630
dmPFC - Model 2	0.2766	0.0975	0.0059	-0.9618	0.1141	0.2766	0.8920	0.4709
dmPFC - Model 3	-0.0365	0.0831	0.6617	-1.0930	-0.1751	-0.0365	0.4884	0.1293
lATL - Model 1	0.2348	0.1090	0.0345	-1.1496	0.0532	0.2348	0.9228	0.4521
lATL - Model 2	-0.0052	0.0846	0.9510	-1.0798	-0.1462	-0.0052	0.5287	0.1634
lATL - Model 3	0.0470	0.0814	0.5657	-0.9872	-0.0887	0.0470	0.5609	0.2093
lCaudate - Model 1	0.0574	0.1083	0.5976	-1.3181	-0.1230	0.0574	0.7409	0.2733
lCaudate - Model 2	0.1670	0.0873	0.0597	-0.9419	0.0215	0.1670	0.7180	0.3410
lCaudate - Model 3	-0.1305	0.0898	0.1509	-1.2721	-0.2802	-0.1305	0.4368	0.0487
lCrblm - Model 1	-0.0313	0.1069	0.7704	-1.3891	-0.2094	-0.0313	0.6434	0.1818
lCrblm - Model 2	0.1983	0.0857	0.0236	-0.8906	0.0555	0.1983	0.7394	0.3692
lCrblm - Model 3	-0.1931	0.0800	0.0184	-1.2101	-0.3265	-0.1931	0.3123	-0.0335
lTPJ - Model 1	0.2557	0.1101	0.0230	-1.1428	0.0723	0.2557	0.9506	0.4752
lTPJ - Model 2	0.2922	0.0832	0.0008	-0.7645	0.1536	0.2922	0.8173	0.4581
lTPJ - Model 3	-0.0574	0.0850	0.5014	-1.1368	-0.1990	-0.0574	0.4790	0.1120
pgACC - Model 1	-0.0052	0.1090	0.9619	-1.3898	-0.1868	-0.0052	0.6828	0.2121
pgACC - Model 2	0.3027	0.0888	0.0011	-0.8251	0.1547	0.3027	0.8631	0.4797
pgACC - Model 3	-0.1200	0.0807	0.1412	-1.1451	-0.2545	-0.1200	0.3893	0.0408
rATL - Model 1	0.2975	0.1101	0.0086	-1.1014	0.1140	0.2975	0.9926	0.5170
rATL - Model 2	0.1252	0.0804	0.1237	-0.8962	-0.0087	0.1252	0.6328	0.2855
rATL - Model 3	-0.0052	0.0823	0.9496	-1.0504	-0.1423	-0.0052	0.5141	0.1588

rCrblm - Model 1	0.1931	0.1211	0.1153	-1.3456	-0.0087	0.1931	0.9577	0.4345
rCrblm - Model 2	0.0887	0.0887	0.3207	-1.0385	-0.0591	0.0887	0.6488	0.2656
rCrblm - Model 3	-0.0992	0.0739	0.1842	-1.0387	-0.2224	-0.0992	0.3677	0.0483
rITG - Model 1	0.1879	0.1156	0.1086	-1.2811	-0.0048	0.1879	0.9178	0.4184
rITG - Model 2	0.1200	0.0789	0.1328	-0.8830	-0.0115	0.1200	0.6184	0.2774
rITG - Model 3	-0.1774	0.0739	0.0190	-1.1169	-0.3007	-0.1774	0.2894	-0.0300
rTPJ - Model 1	0.2505	0.1182	0.0376	-1.2515	0.0535	0.2505	0.9968	0.4862
rTPJ - Model 2	0.3653	0.0863	0.0001	-0.7318	0.2214	0.3653	0.9104	0.5375
rTPJ - Model 3	-0.2296	0.0691	0.0014	-1.1082	-0.3448	-0.2296	0.2069	-0.0917
rvlPFC - Model 1	0.1305	0.1155	0.2626	-1.3375	-0.0621	0.1305	0.8599	0.3608
rvlPFC - Model 2	0.1357	0.0893	0.1330	-0.9987	-0.0131	0.1357	0.6994	0.3137
rvlPFC - Model 3	0.0731	0.0806	0.3678	-0.9512	-0.0613	0.0731	0.5820	0.2338
sgACC - Model 1	-0.0313	0.1139	0.7841	-1.4781	-0.2211	-0.0313	0.6876	0.1957
sgACC - Model 2	-0.0261	0.0781	0.7392	-1.0180	-0.1562	-0.0261	0.4668	0.1296
sgACC - Model 3	0.0209	0.0810	0.7975	-1.0088	-0.1142	0.0209	0.5325	0.1825
vmPFC - Model 1	-0.0209	0.1127	0.8536	-1.4527	-0.2087	-0.0209	0.6906	0.2038
vmPFC - Model 2	0.1409	0.0826	0.0923	-0.9083	0.0033	0.1409	0.6622	0.3055
vmPFC - Model 3	-0.0887	0.0749	0.2400	-1.0401	-0.2135	-0.0887	0.3840	0.0606

Supplemental Figure 3: Comparison of model fit by number of usable runs within social interaction ROIs

As a means to evaluate the decision to include participants with a minimum of two usable functional runs of the experiment, we re-ran RSA for each ROI using participants with a minimum of three (red) and four (blue) usable runs for a side-by-side comparison with results from all participants (grey; who had a minimum of two usable runs) the results. Overall, the results are comparable, suggesting that we were able to obtain reliable estimates even with less data. Mean model fit estimates are presented as bar graphs with uncorrected p-values from Wilcoxon signed-rank tests are displayed under each.

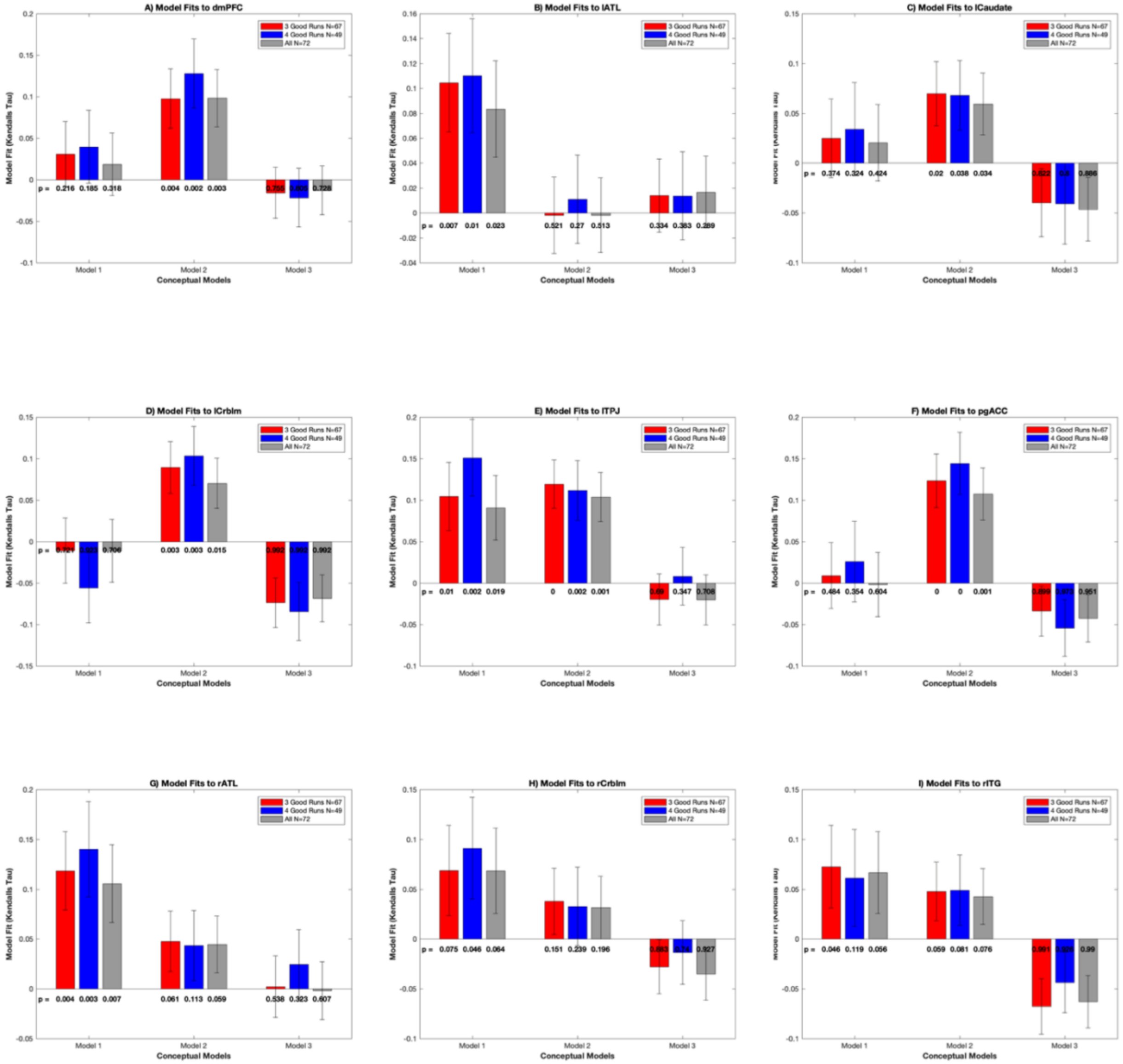
A) Model Fits to dmPFC
B) Model Fits to lATL
C) Model Fits to lCaudate
D) Model Fits to lCrblm
E) Model Fits to lTPJ
F) Model Fits to pgACC
G) Model Fits to rATL
H) Model Fits to rCrblm
I) Model Fits to rITG
3 Good Runs N=67
4 Good Runs N=49
All N=72
Model Fit (Kendalls Tau)
Model 1
Model 2
Model 3
Conceptual Models

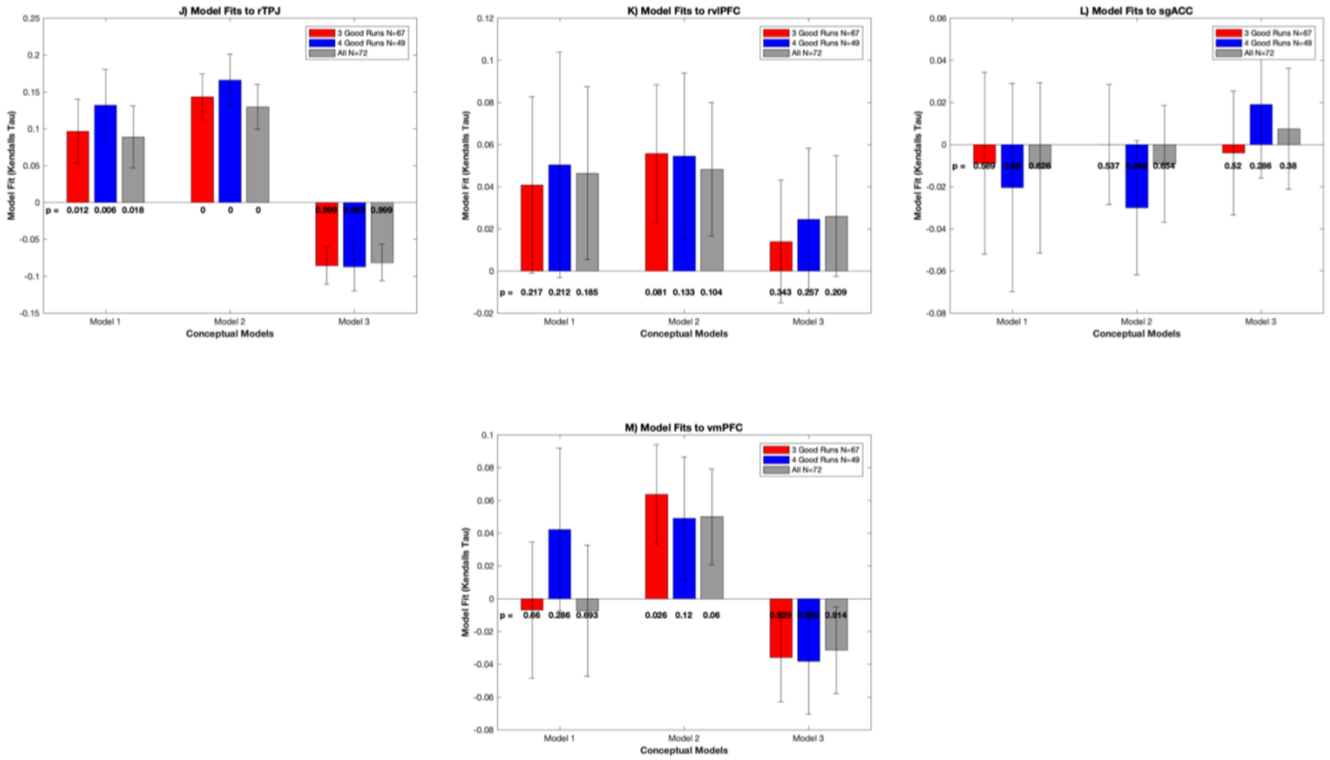

Supplemental Table 9: Follow up regressions to examine between-conditions distances as a way to explain negative relationship between age and Model 2 fit

Follow-up analyses were conducted to assess which between-condition distances were contributing to the negative correlations between age and Model 2 presented in the main text. To this end, we calculated Spearman's rank-order correlation between age and the six between-condition distances for the left caudate, left TPJ, right vlPFC. These were followed up with regression analyses with age as the dependent variable and the six between-condition distances as independent variables to assess whether the findings yielded from the correlations were the driving force even when controlling for the other between-condition distances.

Spearman's Correlations

Variable		Age
1. Age	Spearman's rho	—
	p-value	—
2. lCaudate_CM_CN	Spearman's rho	0.047
	p-value	0.695
3. lCaudate_CM_PM	Spearman's rho	−0.052
	p-value	0.665
4. lCaudate_CM_PN	Spearman's rho	0.120
	p-value	0.317
5. lCaudate_CN_PM	Spearman's rho	−0.201
	p-value	0.091
6. lCaudate_CN_PN	Spearman's rho	−0.078
	p-value	0.516
7. lCaudate_PM_PN	Spearman's rho	0.297*
	p-value	0.011
8. lTPJ_CM_CN	Spearman's rho	0.142
	p-value	0.233
9. lTPJ_CM_PM	Spearman's rho	−0.005
	p-value	0.969
10. lTPJ_CM_PN	Spearman's rho	−0.054
	p-value	0.652
11. lTPJ_CN_PM	Spearman's rho	−0.125
	p-value	0.296
12. lTPJ_CN_PN	Spearman's rho	−0.127
	p-value	0.286
13. lTPJ_PM_PN	Spearman's rho	0.134
	p-value	0.262
14. rvlPFC_CM_CN	Spearman's rho	0.097
	p-value	0.415
15. rvlPFC_CM_PM	Spearman's rho	−0.115
	p-value	0.335
16. rvlPFC_CM_PN	Spearman's rho	−0.259*
	p-value	0.028
17. rvlPFC_CN_PM	Spearman's rho	−0.174
	p-value	0.144
18. rvlPFC_CN_PN	Spearman's rho	0.048
	p-value	0.689
19. rvlPFC_PM_PN	Spearman's rho	0.234*
	p-value	0.048

* $p < .05$, ** $p < .01$, *** $p < .001$

Linear Regression – lCaudate

Model Summary – Age

Model	R	R^2	Adjusted R^2	RMSE
H_0	0.350	0.122	0.041	1.754

Note. Null model includes lCaudate_CM_CN, lCaudate_CM_PM, lCaudate_CM_PN, lCaudate_CN_PM, lCaudate_CN_PN, lCaudate_PM_PN

Coefficients

Model		Unstandardized	Standard Error	Standardized	t	p
H_0	(Intercept)	10.184	1.098		9.274	1.635e−13
	lCaudate_CM_CN	0.636	0.669	0.129	0.951	0.345
	lCaudate_CM_PM	−0.021	0.597	−0.004	−0.034	0.973
	lCaudate_CM_PN	0.292	0.612	0.060	0.477	0.635
	lCaudate_CN_PM	−0.402	0.619	−0.084	−0.649	0.519
	lCaudate_CN_PN	−0.441	0.683	−0.082	−0.646	0.521
	lCaudate_PM_PN	1.535	0.656	0.309	2.338	0.022

Linear Regression – lTPJ

Model Summary – Age

Model	R	R^2	Adjusted R^2	RMSE
H_0	0.301	0.091	0.007	1.785

Note. Null model includes lTPJ_CM_CN, lTPJ_CM_PM, lTPJ_CM_PN, lTPJ_CN_PM, lTPJ_CN_PN, lTPJ_PM_PN

Coefficients

Model		Unstandardized	Standard Error	Standardized	t	p
H_0	(Intercept)	11.201	1.184		9.457	7.836e−14
	lTPJ_CM_CN	0.866	0.687	0.168	1.261	0.212
	lTPJ_CM_PM	−0.093	0.607	−0.020	−0.154	0.878
	lTPJ_CM_PN	−0.124	0.696	−0.024	−0.178	0.859
	lTPJ_CN_PM	−0.764	0.665	−0.147	−1.149	0.255
	lTPJ_CN_PN	−0.836	0.664	−0.170	−1.260	0.212
	lTPJ_PM_PN	0.738	0.694	0.142	1.064	0.291

Linear Regression – rvlPFC

Model Summary – Age

Model	R	R^2	Adjusted R^2	RMSE
H_0	0.433	0.188	0.113	1.687

Note. Null model includes rvlPFC_CM_CN, rvlPFC_CM_PM, rvlPFC_CM_PN, rvlPFC_CN_PM, rvlPFC_CN_PN, rvlPFC_PM_PN

Coefficients

Model		Unstandardized	Standard Error	Standardized	t	p
H_0	(Intercept)	11.311	1.080		10.474	1.366e−15
	rvlPFC_CM_CN	0.420	0.599	0.085	0.702	0.485
	rvlPFC_CM_PM	0.113	0.711	0.022	0.159	0.874
	rvlPFC_CM_PN	−1.623	0.617	−0.312	−2.629	0.011
	rvlPFC_CN_PM	−1.153	0.600	−0.221	−1.923	0.059
	rvlPFC_CN_PN	0.399	0.652	0.084	0.612	0.543
	rvlPFC_PM_PN	1.253	0.651	0.247	1.925	0.059

Section 5: Whole-Brain Searchlight Analysis

Supplemental Methods 4: Searchlight analysis

Exploratory searchlight analyses were performed using CoSMoMVPA (Oosterhof et al., 2016) for each of the three models to reveal model fits in brain regions that were not previously considered. This approach involves extracting local patterns of brain activity throughout each participant's brain. A sphere of approximately 100 voxels was drawn around each gray matter voxel, and the voxel-wise t-values are extracted for each condition. A neural RDM was created by calculating the Euclidean distance between each pair of conditions, and model fit between the neural and model RDMs was estimated using CoSMoMVPA's implementation of Kendall's Tau-a. After performing a Fisher z transformation, the z-value was assigned to the center voxel, yielding whole-brain maps of model fit estimates throughout the brain. One-sample t-tests were calculated on these subject-level maps for each model using AFNI's 3dttest++ to determine significant model fits across our sample. For all group-level analyses, the cluster correction value was calculated automatically by using the -Clustsim option in AFNI's 3dttest++, which sends the input volumes directly to the 3dClustSim program after simulating the noise volumes by randomizing and permuting the input datasets. This model-free approach is more accurate at controlling the false positive rate compared to simulating noise using a mixed-model estimation of the autocorrelation function (Cox et al., 2017). This yielded a voxel-wise threshold of $p < .001$ and cluster extent of $k = 117\text{-}12451$ to achieve a whole-brain familywise error rate of $\alpha = 0.05$

when nearest neighbor cluster definitions are set to 3 (i.e., voxel faces, edges, and corners touching count as part of a contiguous cluster).

Supplemental Figure 4: Searchlight results for Models 1 & 2

Searchlight results for Model 1 in red (A) and Model 2 in green (B). Clusters that show significantly greater fit for Model 2 than Model 1 are displayed in blue.

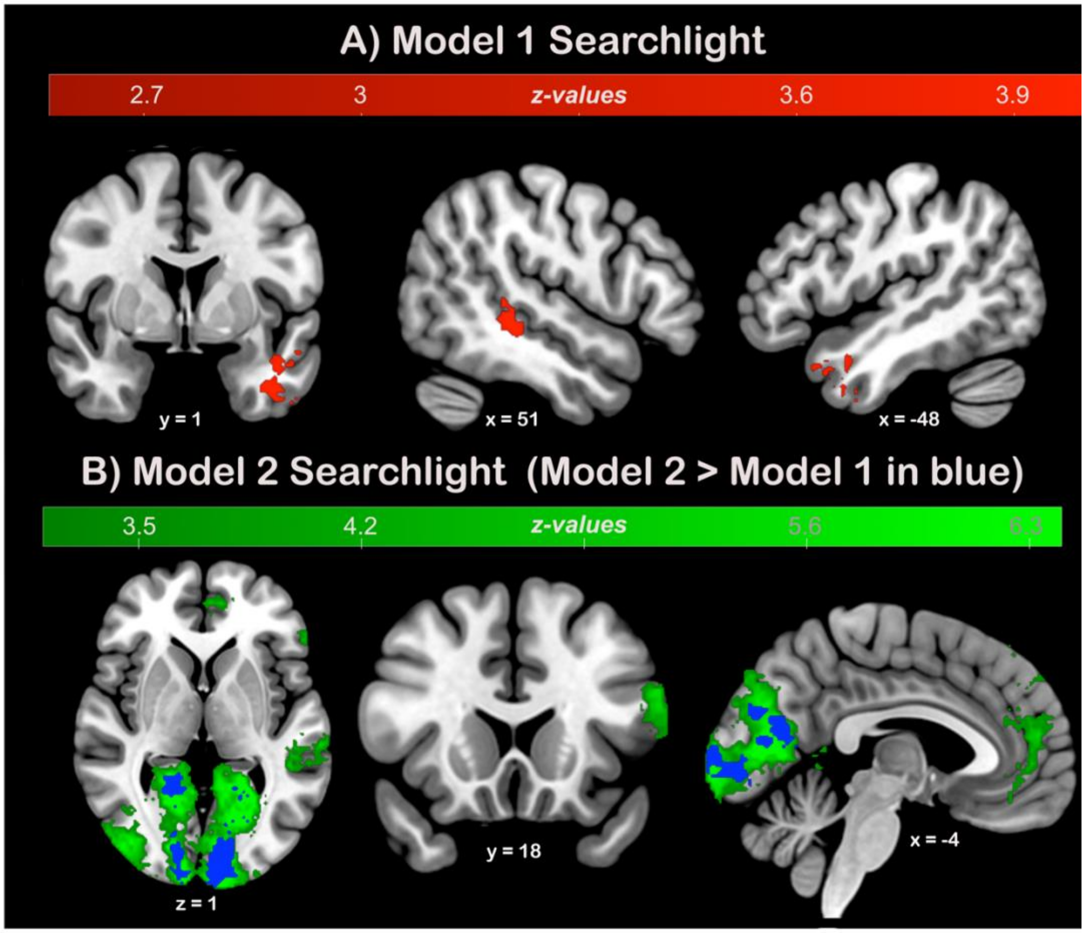

Supplemental Table 10: Searchlight results for Models 1 & 2

Cluster showing significant fit to Model 1 (A) and Model 2 (B).

**A) Model 1 Searchlight *p* < .001, k = 124**				**MNI Coordinates (center of mass)**		
**Region**	**Hemisphere**	**Cluster Size (voxels)**	**Peak Z**	**x**	**y**	**z**
Anterior Temporal Lobe*	L	222	4.26	-46	4	-32
Posterior Superior Temporal Sulcus*	R	156	4.35	48	-33	-1
**Not significant at cluster corrected threshold. Only for exploration: p < .005, k = 150*						

**B) A) Model 2 Searchlight *p* < .001, k = 117**				**MNI Coordinates (center of mass)**		
**Region**	**Hemisphere**	**Cluster Size (voxels)**	**Peak Z**	**x**	**y**	**z**
Visual Cortex	R	12443	7.29	3	-76	5
Dorsal to Ventral Medial Prefrontal Cortex	L	513	4.65	-5	48	14
Angular Gyrus/TPJ	R	420	4.78	56	-49	25
Posterior Superior Temporal Sulcus	L	348	4.37	-57	-33	1
Supramarginal Gyrus/TPJ	L	295	5.14	-57	-55	27
Inferior Frontal Gyrus (triangularis)	L	272	4.84	-56	22	12
Superior Frontal Gyrus/Frontal Pole	L	263	4.57	-7	46	44

Section 6: Reanalysis After Excluding Incorrectly Answered Trials

Supplemental Methods 5: Reanalysis excluding incorrect trials

The results reported in the main text were from subject-level models that included all trials, whether or not they were answered correctly. The rationale was that participants may have engaged in the process of interest (e.g., mentalizing) even if they chose the incorrect response option. Moreover, some of the trial items had ambiguously correct response options (see Supplemental Table 1 for all items). To evaluate the impact of including all trials (including "incorrect") in our analyses, we re-analyzed the data after removing the trials that were incorrectly answered based on our *a priori* choice of which answers were correct. This required slightly different modeling procedures for the subject-level modeling of the fMRI data: 1) an extra regressor of no interest for incorrect trials was added; 2) because some participants had runs with no incorrect trials, meaning there was no regressor for that condition, we modeled all runs together in this set of analyses. This is different from the main set of analyses, which were modeled at the run level and combined for subsequent analyses to evaluate run-level effects and assess reliability.

Supplemental Figure 5: Social interaction ROIs

Similar to the results of the primary analyses, bilateral ATL and right TPJ exhibited very strong evidence for Model 1 fit as indicated by the intercept estimate lying beyond the 97.5% quantile level in the positive domain, although the intercept for the left TPJ now lies in the 95%-97.5% quantile range, which is still indicative of strong evidence. Green = 97.5% quantile or more; orange = 95%-97.5% quantile range; grey = 90%-95% quantile range.

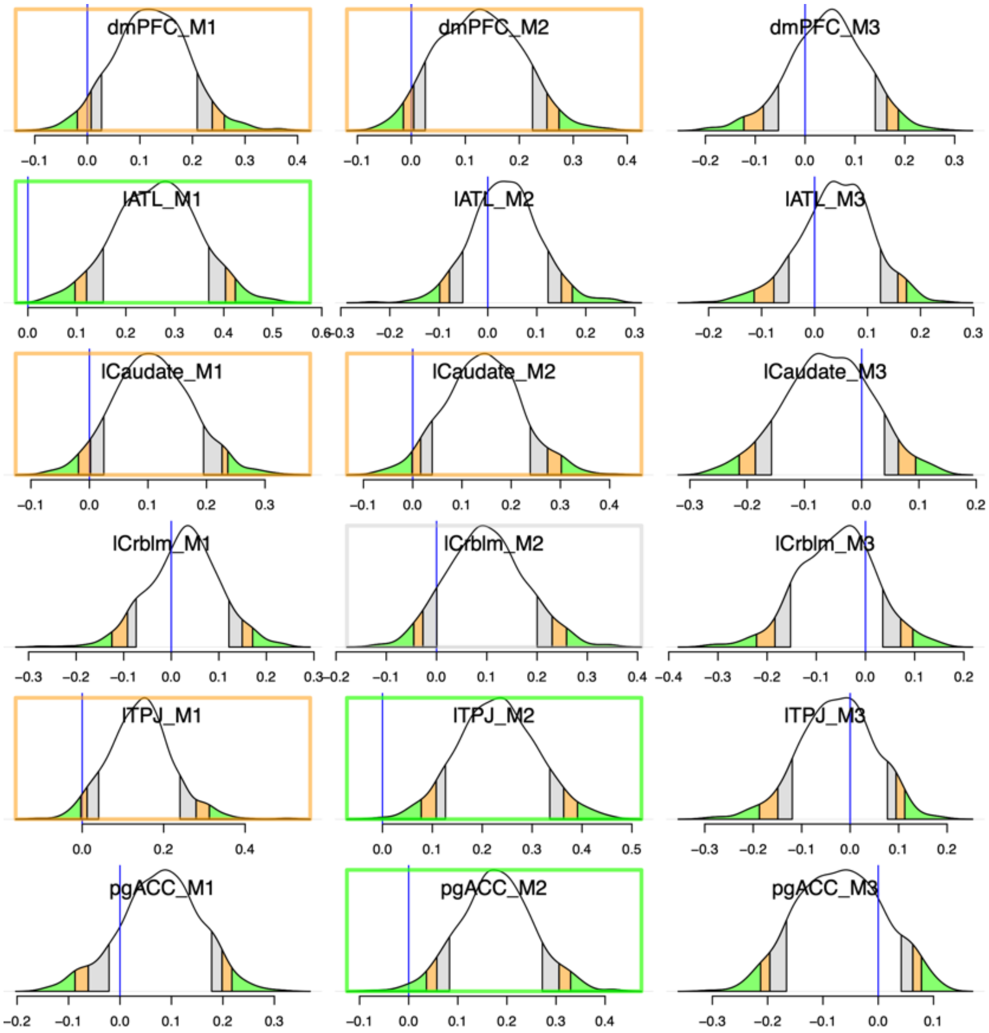
dmPFC_M1
dmPFC_M2
dmPFC_M3
lATL_M1
lATL_M2
lATL_M3
lCaudate_M1
lCaudate_M2
lCaudate_M3
lCrblm_M1
lCrblm_M2
lCrblm_M3
lTPJ_M1
lTPJ_M2
lTPJ_M3
pgACC_M1
pgACC_M2
pgACC_M3

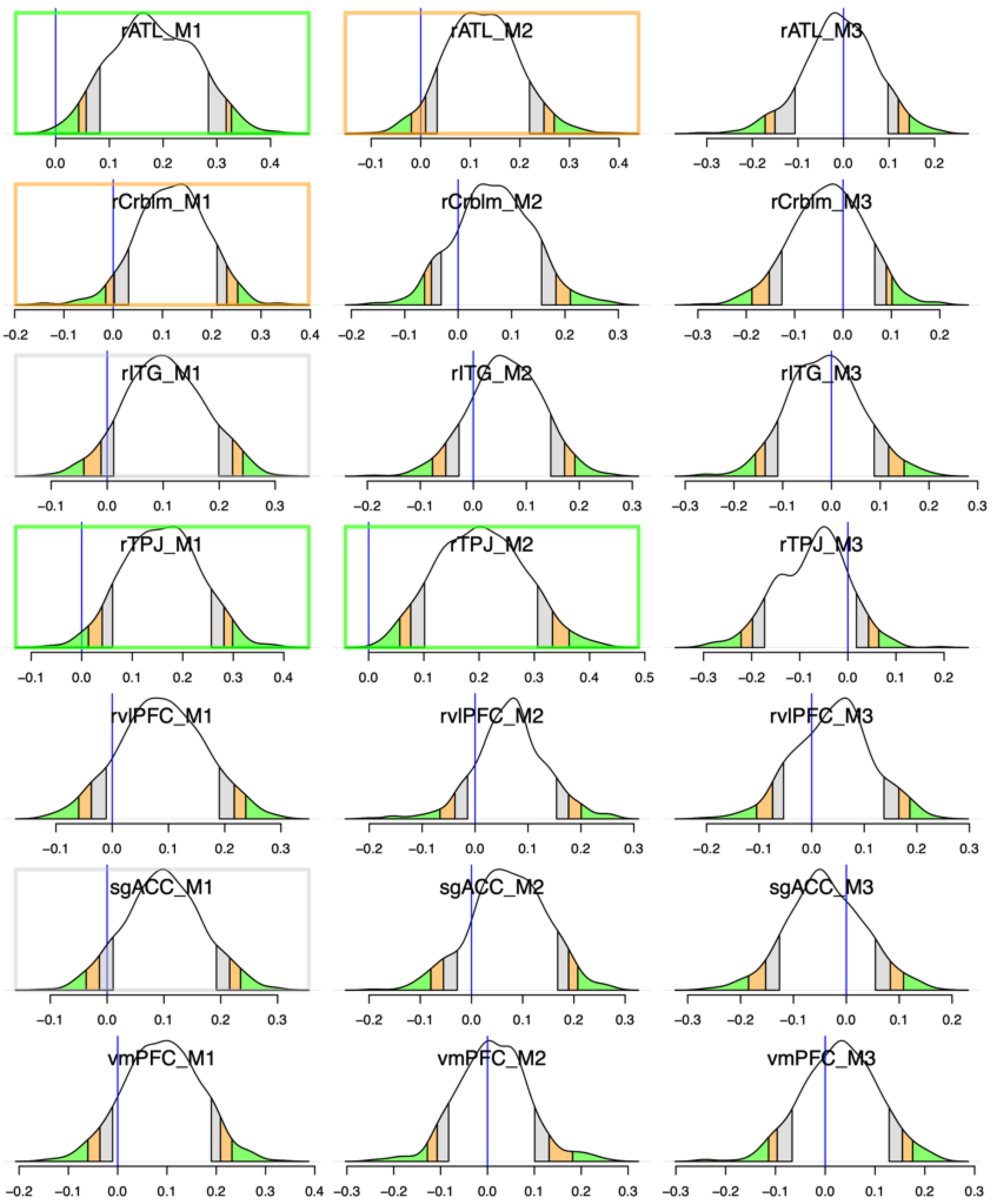

Supplemental Figure 6: Searchlight analysis

Supplemental searchlight analysis using subject-level models that did not include incorrectly responded trials followed the same procedure as the searchlight analyses using the subject level models of the primary set of analyses (Supplemental Methods 4). Similar to before, whole-brain Model 1 fit is presented using exploratory, uncorrected threshold, and reveals only a cluster in the left ATL. Searchlight results for Model 1 in red (A) and Model 2 in green (B).

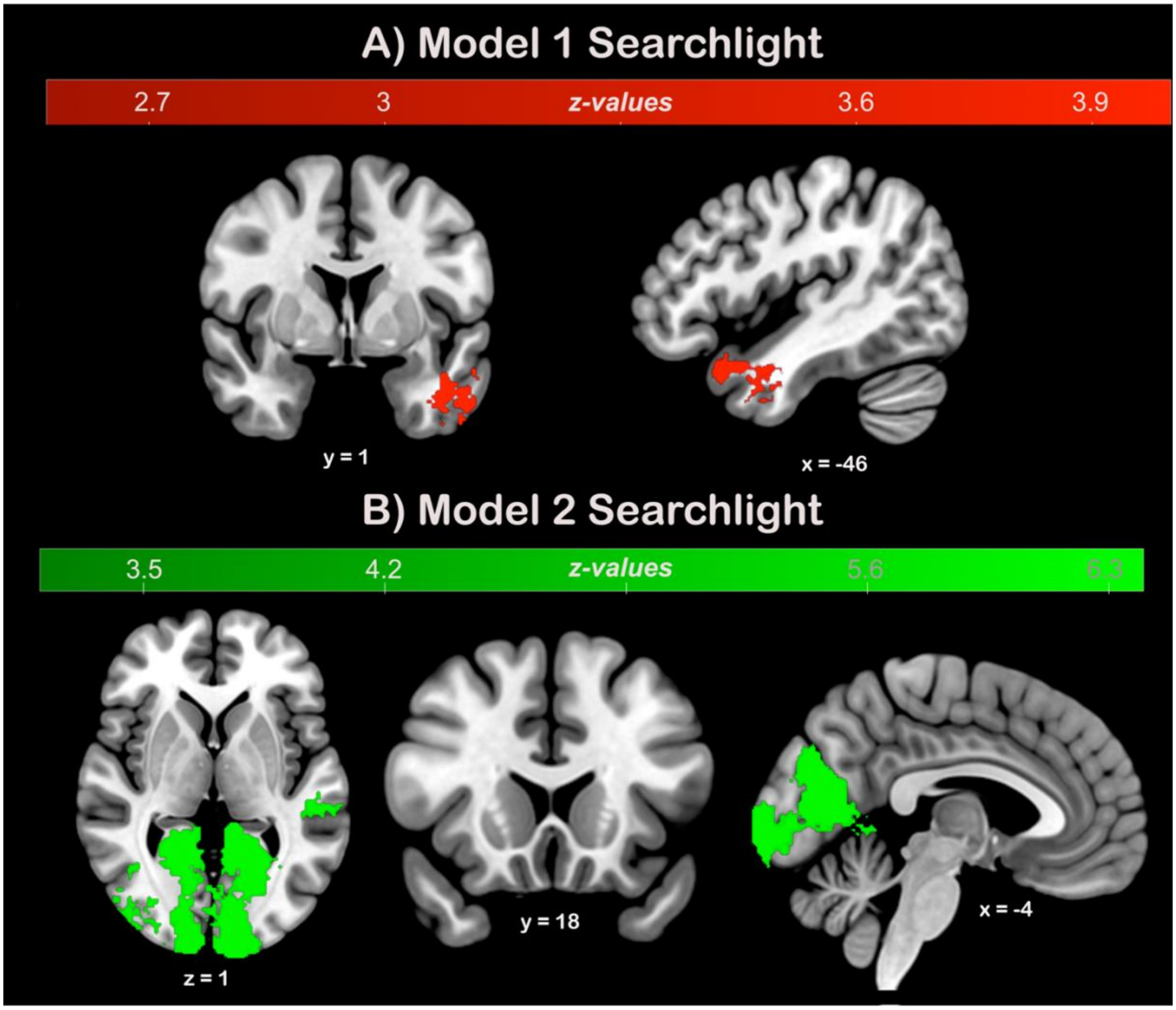

Supplemental Table 11: Searchlight clusters

Cluster showing significant fit to Model 1 (A) and Model 2 (B).

**A) Model 1 Searchlight** ***p*** **< .001, k = 150**				**MNI Coordinates (center of mass)**		
**Region**	**Hemisphere**	**Cluster Size (voxels)**	**Peak Z**	**x**	**y**	**z**
Anterior Temporal Lobe*	L	517	4.14	-45	6	-32
**Not significant at cluster corrected threshold. Only for exploration: p < .005, k = 150*						

B) A) Model 2 Searchlight, *p* < .001, k = 126

Region	Hemisphere	Cluster Size (voxels)	Peak Z	MNI Coordinates (center of mass) x	y	z
Visual Cortex	R	9556	6.76	4	-76	5
Supramarginal Gyrus/TPJ	L	271	5.06	-54	-57	25
Posterior Superior Temporal Sulcus	L	174	4.55	-55	-31	1
Angular Gyrus/TPJ	R	127	4.64	48	-50	30

Supplemental References

Abraham, Alexandre, Fabian Pedregosa, Michael Eickenberg, Philippe Gervais, Andreas Mueller, Jean Kossaifi, Alexandre Gramfort, Bertrand Thirion, and Gael Varoquaux. 2014. “Machine Learning for Neuroimaging with Scikit-Learn.” *Frontiers in Neuroinformatics* 8. https://doi.org/10.3389/fninf.2014.00014.

Alkire, D., Levitas, D., Warnell, K. R., & Redcay, E. (2018). Social interaction recruits mentalizing and reward systems in middle childhood. *Human Brain Mapping*, *39*(10), 3928–3942. https://doi.org/10.1002/hbm.24221

Avants, B.B., C.L. Epstein, M. Grossman, and J.C. Gee. 2008. “Symmetric Diffeomorphic Image Registration with Cross-Correlation: Evaluating Automated Labeling of Elderly and Neurodegenerative Brain.” *Medical Image Analysis* 12 (1): 26–41. https://doi.org/10.1016/j.media.2007.06.004.

Behzadi, Yashar, Khaled Restom, Joy Liau, and Thomas T. Liu. 2007. “A Component Based Noise Correction Method (CompCor) for BOLD and Perfusion Based fMRI.” *NeuroImage* 37 (1): 90–101. https://doi.org/10.1016/j.neuroimage.2007.04.042.

Chen, G., Taylor, P. A., Stoddard, J., Cox, R. W., Bandettini, P. A., & Pessoa, L. (2021). *Dichotomous thinking and informational waste in neuroimaging* (p. 2021.05.09.443246). https://doi.org/10.1101/2021.05.09.443246

Chen, G., Xiao, Y., Taylor, P. A., Rajendra, J. K., Riggins, T., Geng, F., Redcay, E., & Cox, R. W. (2019). Handling Multiplicity in Neuroimaging Through Bayesian Lenses with Multilevel Modeling. *Neuroinformatics*, *17*(4), 515–545. https://doi.org/10.1007/s12021-018-9409-6

Cox, Robert W., and James S. Hyde. 1997. “Software Tools for Analysis and Visualization of fMRI Data.” *NMR in Biomedicine* 10 (4-5): 171–78. https://doi.org/10.1002/(SICI)1099-1492(199706/08)10:4/5<171::AID-NBM453>3.0.CO;2-L.

Dale, Anders M., Bruce Fischl, and Martin I. Sereno. 1999. “Cortical Surface-Based Analysis: I. Segmentation and Surface Reconstruction.” *NeuroImage* 9 (2): 179–94. https://doi.org/10.1006/nimg.1998.0395.

Egger, H. L., Pine, D. S., Nelson, E., Leibenluft, E., Ernst, M., Towbin, K. E., & Angold, A. (2011). The NIMH Child Emotional Faces Picture Set (NIMH‐ChEFS): a new set of children's facial emotion stimuli. *International journal of methods in psychiatric research*, *20*(3), 145-156.

Esteban, Oscar, Ross Blair, Christopher J. Markiewicz, Shoshana L. Berleant, Craig Moodie, Feilong Ma, Ayse Ilkay Isik, et al. 2018. “FMRIPrep.” *Software*. Zenodo. https://doi.org/10.5281/zenodo.852659.

Esteban, Oscar, Christopher Markiewicz, Ross W Blair, Craig Moodie, Ayse Ilkay Isik, Asier Erramuzpe Aliaga, James Kent, et al. 2018. “fMRIPrep: A Robust Preprocessing Pipeline for Functional MRI.” *Nature Methods*. https://doi.org/10.1038/s41592-018-0235-4.

Evans, AC, AL Janke, DL Collins, and S Baillet. 2012. “Brain Templates and Atlases.” *NeuroImage* 62 (2): 911–22. https://doi.org/10.1016/j.neuroimage.2012.01.024.

Fonov, VS, AC Evans, RC McKinstry, CR Almli, and DL Collins. 2009. “Unbiased Nonlinear Average Age-Appropriate Brain Templates from Birth to Adulthood.” *NeuroImage* 47, Supplement 1: S102. https://doi.org/10.1016/S1053-8119(09)70884-5.

Gorgolewski, K., C. D. Burns, C. Madison, D. Clark, Y. O. Halchenko, M. L. Waskom, and S. Ghosh. 2011. “Nipype: A Flexible, Lightweight and Extensible Neuroimaging Data Processing Framework in Python.” *Frontiers in Neuroinformatics* 5: 13. https://doi.org/10.3389/fninf.2011.00013.

Gorgolewski, Krzysztof J., Oscar Esteban, Christopher J. Markiewicz, Erik Ziegler, David Gage Ellis, Michael Philipp Notter, Dorota Jarecka, et al. 2018. “Nipype.” *Software*. Zenodo. https://doi.org/10.5281/zenodo.596855.

Greve, Douglas N, and Bruce Fischl. 2009. “Accurate and Robust Brain Image Alignment Using Boundary-Based Registration.” *NeuroImage* 48 (1): 63–72. https://doi.org/10.1016/j.neuroimage.2009.06.060.

Huntenburg, Julia M. 2014. “Evaluating Nonlinear Coregistration of BOLD EPI and T1w Images.” Master’s Thesis, Berlin: Freie Universität. http://hdl.handle.net/11858/00-001M-0000-002B-1CB5-A.

Jenkinson, Mark, Peter Bannister, Michael Brady, and Stephen Smith. 2002. “Improved Optimization for the Robust and Accurate Linear Registration and Motion Correction of Brain Images.” *NeuroImage* 17 (2): 825–41. https://doi.org/10.1006/nimg.2002.1132.

Klein, Arno, Satrajit S. Ghosh, Forrest S. Bao, Joachim Giard, Yrjö Häme, Eliezer Stavsky, Noah Lee, et al. 2017. “Mindboggling Morphometry of Human Brains.” *PLOS Computational Biology* 13 (2): e1005350. https://doi.org/10.1371/journal.pcbi.1005350.

Lanczos, C. 1964. “Evaluation of Noisy Data.” *Journal of the Society for Industrial and Applied Mathematics Series B Numerical Analysis* 1 (1): 76–85. https://doi.org/10.1137/0701007.

Power, Jonathan D., Anish Mitra, Timothy O. Laumann, Abraham Z. Snyder, Bradley L. Schlaggar, and Steven E. Petersen. 2014. “Methods to Detect, Characterize, and Remove Motion Artifact in Resting State fMRI.” *NeuroImage* 84 (Supplement C): 320–41. https://doi.org/10.1016/j.neuroimage.2013.08.048.

Pruim, Raimon H. R., Maarten Mennes, Daan van Rooij, Alberto Llera, Jan K. Buitelaar, and Christian F. Beckmann. 2015. “ICA-AROMA: A Robust ICA-Based Strategy for Removing Motion Artifacts from fMRI Data.” *NeuroImage* 112 (Supplement C): 267–77. https://doi.org/10.1016/j.neuroimage.2015.02.064.

Satterthwaite, Theodore D., Mark A. Elliott, Raphael T. Gerraty, Kosha Ruparel, James Loughead, Monica E. Calkins, Simon B. Eickhoff, et al. 2013. “An improved framework for confound regression and filtering for control of motion artifact in the preprocessing of resting-state functional connectivity data.” *NeuroImage* 64 (1): 240–56. https://doi.org/10.1016/j.neuroimage.2012.08.052.

Treiber, Jeffrey Mark, Nathan S. White, Tyler Christian Steed, Hauke Bartsch, Dominic Holland, Nikdokht Farid, Carrie R. McDonald, Bob S. Carter, Anders Martin Dale, and Clark C. Chen. 2016. “Characterization and Correction of Geometric Distortions in 814 Diffusion Weighted Images.” *PLOS ONE* 11 (3): e0152472. https://doi.org/10.1371/journal.pone.0152472.

Tustison, N. J., B. B. Avants, P. A. Cook, Y. Zheng, A. Egan, P. A. Yushkevich, and J. C. Gee. 2010. “N4ITK: Improved N3 Bias Correction.” *IEEE Transactions on Medical Imaging* 29 (6): 1310–20. https://doi.org/10.1109/TMI.2010.2046908.

Wang, Sijia, Daniel J. Peterson, J. C. Gatenby, Wenbin Li, Thomas J. Grabowski, and Tara M. Madhyastha. 2017. “Evaluation of Field Map and Nonlinear Registration Methods for Correction

of Susceptibility Artifacts in Diffusion MRI." *Frontiers in Neuroinformatics* 11. https://doi.org/10.3389/fninf.2017.00017.

Zhang, Y., M. Brady, and S. Smith. 2001. "Segmentation of Brain MR Images Through a Hidden Markov Random Field Model and the Expectation-Maximization Algorithm." *IEEE Transactions on Medical Imaging* 20 (1): 45–57. https://doi.org/10.1109/42.906424.
